# Can we have a second helping? A preregistered direct replication study on the neurobiological mechanisms underlying self‐control

**DOI:** 10.1002/hbm.26065

**Published:** 2022-09-09

**Authors:** Christin Scholz, Hang‐Yee Chan, Russell A. Poldrack, Denise T. D. de Ridder, Ale Smidts, Laura Nynke van der Laan

**Affiliations:** ^1^ Amsterdam School of Communication Research University of Amsterdam Amsterdam The Netherlands; ^2^ Department of Psychology Stanford University Stanford California USA; ^3^ Department of Social, Health and Organisational Psychology Utrecht University Utrecht The Netherlands; ^4^ Rotterdam School of Management Erasmus University Rotterdam Rotterdam The Netherlands; ^5^ Department of Communication and Cognition Tilburg University Tilburg The Netherlands

**Keywords:** dlPFC, fMRI, replication, self‐control, vmPFC

## Abstract

Self‐control is of vital importance for human wellbeing. Hare et al. (2009) were among the first to provide empirical evidence on the neural correlates of self‐control. This seminal study profoundly impacted theory and empirical work across multiple fields. To solidify the empirical evidence supporting self‐control theory, we conducted a preregistered replication of this work. Further, we tested the robustness of the findings across analytic strategies. Participants underwent functional magnetic resonance imaging while rating 50 food items on healthiness and tastiness and making choices about food consumption. We closely replicated the original analysis pipeline and supplemented it with additional exploratory analyses to follow‐up on unexpected findings and to test the sensitivity of results to key analytical choices. Our replication data provide support for the notion that decisions are associated with a value signal in ventromedial prefrontal cortex (vmPFC), which integrates relevant choice attributes to inform a final decision. We found that vmPFC activity was correlated with goal values regardless of the amount of self‐control and it correlated with both taste and health in self‐controllers but only taste in non‐self‐controllers. We did not find strong support for the hypothesized role of left dorsolateral prefrontal cortex (dlPFC) in self‐control. The absence of statistically significant group differences in dlPFC activity during successful self‐control in our sample contrasts with the notion that dlPFC involvement is required in order to effectively integrate longer‐term goals into subjective value judgments. Exploratory analyses highlight the sensitivity of results (in terms of effect size) to the analytical strategy, for instance, concerning the approach to region‐of‐interest analysis.

## Graphical abstract

To solidify the empirical evidence supporting self‐control theory, we conducted a preregistered replication of the seminal study of Hare et al. (2009). Our replication data provide support for the notion that decisions are associated with a value signal in ventromedial prefrontal cortex but no strong support was found for the hypothesized role of left dorsolateral prefrontal cortex in self‐control. Exploratory analyses highlight the sensitivity of results (in terms of effect size) to the analytical strategy, for instance, concerning the approach to region‐of‐interest analysis.

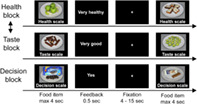

## 1 INTRODUCTION

Self‐control, the ability to prioritize long‐term goals over immediate urges, is of vital importance for human wellbeing (de Ridder et al., 2012; Inzlicht et al., 2021; Metcalfe & Mischel, 1999). Uncovering the neural underpinnings of self‐control facilitates a full understanding of its mechanisms and, thus, contributes to improvement in the prediction of (failure of) self‐control and the development of effective interventions. A seminal study by Hare et al. (2009) was among the first to identify the neural correlates of self‐control in the context of food consumption. This study showed that a food's value is encoded in the ventromedial prefrontal cortex (vmPFC) and that successful self‐control entails modulation of the vmPFC signal by activity in left dorsolateral prefrontal cortex (left dlPFC). This work conducted by Hare et al. has profoundly impacted interdisciplinary science, with over 2000 citations on Google Scholar.^1^ In the theoretical realm, insights about the neural correlates of self‐control derived from this seminal work reinforced interest in neurobiologically informed value‐based choice models of self‐control (Berkman et al., 2017), which describe self‐control as choices that are driven by differential value responses to multiple choice attributes (e.g., food consumption choices based on the healthiness and tastiness of the available food). Despite its impact, the work conducted by Hare et al. (2009) has never been replicated. As is true for many other studies relying on functional magnetic resonance imaging (fMRI), the reliability of the findings reported by Hare et al. requires independent confirmation given important limitations of the original work including a small sample size (Button et al., 2013; Yeung, 2018) and potential effects of analytical and experimental flexibility (Botvinik‐Nezer et al., 2020). To solidify the empirical evidence supporting self‐control theory, we aimed to directly replicate the study reported by Hare et al. (hereafter referred to as the *original study*). In addition, we aimed to determine the robustness of the findings across analytic strategies.

### 1.1 A neurobiological, value‐based decision model of self‐control

Self‐control has been widely studied since its conception approximately 50 years ago (de Ridder et al., 2012; Inzlicht et al., 2021; Metcalfe & Mischel, 1999). The definition of self‐control (Gillebaart, 2018; Milyavskaya et al., 2019), the models and theories that explain it (Inzlicht et al., 2021), the methodology used to assess it (Duckworth & Kern, 2011; Enkavi et al., 2019), and its distinction from related concepts like self‐regulation and cognitive control (Eisenberg et al., 2019), have been under ongoing scrutiny and debate. Facilitated by the development of advanced measurement techniques like fMRI and Ecological Momentary Assessment (EMA), endeavors in both the field of neuroscience and psychology have vastly proliferated models of self‐control. Despite these rapid developments, the field has faced several major setbacks due to nonreplication of highly influential (behavioral) findings (Hagger et al., 2016).

By identifying neural correlates of self‐control in the context of food consumption, the original study reinforced interest in a neurobiologically informed, value‐based choice model of self‐control (Berkman et al., 2017). Choice models assume that the subjective value of an option equals “the weighted sum of choice‐relevant attribute values […] which can vary by person, context and time” (Berkman et al., 2017, p. 3). More specifically, the findings of the original study gave rise to a single process model of self‐control whereby self‐control is the outcome of multiple simple value calculations (Kelley et al., 2015).

In the original study, participants' brains were scanned using fMRI while they made consumption decisions about foods varying in two choice‐relevant attributes, namely the immediate reward (tastiness) and the extent to which the food was in line with participants' long‐term goal of losing weight (healthiness). In participants who were relatively successful self‐controllers (SC; based on their choices during the study) vmPFC activation correlated with both health and taste ratings, while in nonsuccessful SCs vmPFC activation correlated only with tastiness. This supported the view that vmPFC has a critical position of arbiter that calculates and integrates the costs and benefits of several factors (immediate rewards and long‐term goals) which are subjectively relevant to the decision‐maker, before selecting the option with the greatest value (Levy & Glimcher, 2011; Levy & Glimcher, 2012; Rangel, 2013). In addition, the original study demonstrated greater dlPFC activation in successful self‐control trials and a psychophysiological interaction (PPI) analysis showed that the dlPFC was (indirectly), negatively, functionally connected to the vmPFC. Combined, these findings led to the conclusion that the extent to which the dlPFC modulates vmPFC activity determines to what extent higher order factors, such as long‐term health goals, are incorporated into the vmPFC signal, and thus whether self‐control is successfully exercised.

### 1.2 Replication in neuroimaging

Although growing concern about the reproducibility of scientific findings (the “replication crisis”) has led to several replication projects in fields like social psychology (Open Science Collaboration, 2015) and biomedical research (Prinz et al., 2011), replication remains uncommon in neuroscience. This is partly due to the high monetary and time‐investment costs of fMRI studies. Yet, current scientific consensus generally highlights the value of replication and other transparent practices in determining and increasing the robustness of research findings (Zwaan et al., 2018). Empirical evidence suggests that such developments are needed urgently. For instance, several meta‐analyses of fMRI studies have shown low to moderate overlap in findings across different fMRI studies on the same issue (van der Laan et al., 2011; van Meer et al., 2015). One source of unreliability are the traditionally small sample sizes in neuroimaging research which lead to underpowered statistical analyses, a reduced likelihood of detecting relevant effects, and increased the reporting of overestimated effect sizes (Button et al., 2013; Poldrack et al., 2017). Another key concern in neuroimaging is the high degree of analytical flexibility, which can yield different results while analyzing the same data using different, but similarly valid analysis strategies (Botvinik‐Nezer et al., 2020; Poldrack & Poline, 2015). Finally, it is important to validate neuroimaging findings because numerous studies rely on hypothesis‐driven region‐of‐interest (ROI) analyses (Baumgartner et al., 2011). Although limiting the analyses to well informed ROIs reduces the risk of “fishing expeditions,” ROI definitions are often based on somewhat arbitrary decisions and subsequent analyses employ more liberal statistical thresholds for these ROIs which increases the chance to detect false positives. Thus, it is crucial that the function of such a priori hypothesized areas is well established.

### 1.3 Impact of the original study

The original study had implications not limited to the study of self‐control. The results have been used to support hypotheses concerning the role of the vmPFC (Diekhof et al., 2012; Lim et al., 2011; Smith et al., 2014), the dlPFC (Hayashi et al., 2013; Hollmann et al., 2012), or both (Baumgartner et al., 2011; Sokol‐Hessner et al., 2012) in fMRI studies on topics ranging from consumer choice (Enax et al., 2015; Steinbeis et al., 2016) to social rewards (Smith et al., 2014) and risk‐taking (Losecaat Vermeer et al., 2014). For several of these studies, the original study was used to justify the vmPFC and dlPFC as ROIs. Moreover, it has not only served as a basis for functional neuroimaging studies, but also for studies that investigated brain anatomy and connectivity (Qiu et al., 2014; Smith et al., 2014; Steinbeis et al., 2016; Wang et al., 2016) (e.g., voxel‐based morphometry, diffusion tensor imaging, correlational connectivity) underlying the identified mechanisms. Also, studies with techniques that temporarily stimulate or disrupt brain function were designed to study the role of the vmPFC and dlPFC (e.g., transcranial magnetic stimulation) (Baumgartner et al., 2011; Camus et al., 2009; Kekic et al., 2014). Furthermore, results have been used to interpret brain activation during rest (resting‐state fMRI) (Camchong et al., 2013; Chen et al., 2016) and even hypotheses concerning behavioral outcomes were derived from the neural findings (Trendel & Werle, 2016). Finally, the findings have been used to explain results of animal studies (Jentsch et al., 2010).

### 1.4 Current study

As illustrated above, the original study has been used extensively for hypothesis building and interpretation of findings in a wide range of topics in decision‐making, not limited to self‐control. To establish the validity of the models, it is important that the findings they build on are replicated, thereby contributing to the development of neurobiologically underpinned models of human behavior. Importantly, the original study has never been directly replicated. Adapted versions of the task used by Hare et al. have been applied to different (i.e., obese, eating disordered) populations, however, these studies have not consistently shown similar effects as in the original study (Foerde et al., 2015; Medic et al., 2016), which is not unexpected as these clinical populations differ in several aspects (e.g., psychological traits; reward sensitivity (Frank et al., 2018; Meng et al., 2020)) from normal‐weight, noneating‐disordered individuals. Therefore, the first goal of the current study is to directly replicate the original study. To this end, our first research question is identical to the research question of the original study: What are the neurobiological underpinnings of self‐control in decision‐making and how do these neural mechanisms differ between successful and unsuccessful SC? To answer this question, the following four original hypotheses were tested (quoted from Hare et al., 2009, p. 646).

Hypothesis 1 *[A]ctivity in vmPFC should be correlated with participants' goal values regardless of whether or not they exercise self‐control*.

Hypothesis 2 *[A]ctivity in the vmPFC should reflect the health ratings in (participants who are relatively successful SC [i.e., the SC group]) but not (participants who are relatively unsuccessful SC [i.e., the non‐self‐controllers [NSC] group])*.

Hypothesis 3 *[T]he dlPFC should be more active during successful than failed self‐control trials*.

Hypothesis 4 *dlPFC and vmPFC should exhibit functional connectivity during self‐control trials*.

As mentioned above, fMRI data analysis is linked to a large degree of analytical flexibility, so that different, but similarly valid approaches to one data set may lead to different results and conclusions (Botvinik‐Nezer et al., 2020). For this reason, the second goal of this project is to assess if the results of the original study are robust across different analysis pipelines. To this end, we add several analysis branches to the direct replication of the originally reported analysis pipelines to further investigate the impact of important choices made in the original study. Some of the additional methodologically driven analysis branches are motivated by novel developments in what is considered to be “best practice” in fMRI data analysis since the original study was published (Nichols et al., 2017; Smeets et al., 2019). For instance, we assess the impact of the ROI analysis approach on the results. The authors of the original study chose to test some key hypotheses based on neural activity extracted from single peak voxels (identified per subject) within an ROI mask, thus focusing their statistical tests on the question of whether participants, on average, show a hypothesized relationship in neural activity within at least one voxel located in a larger region. An alternative approach is to average signals from all voxels within an ROI for each participant to test the involvement of a larger, static region rather than one specific voxel the location of which varies across individuals. This latter approach also feeds more easily into follow‐up research since it provides empirical support for the involvement of a specific, anatomically identifiable ROI without requiring future researchers to run localizer tasks for the identification of individual peak voxels. In addition, the original study defined functional ROIs based on data from the same sample and task examined during later ROI analysis. Since the publication of the original study, novel tools (e.g., Neurosynth which provides meta‐analytic maps of regions associated with specific psychological processes;  Yarkoni et al., 2011)) have become available that allow easier access to other, independently identified ROIs, not informed and potentially biased by the current data set. The selection of (functional) ROIs is nontrivial, but highly important (Poldrack, 2007), as ROIs are reused frequently by follow‐up research and differing selection choices may or may not lead to contradictory conclusions about the data. One key danger that should be avoided in the selection of nonindependent ROIs (i.e., ROIs defined based on the data that is being analyzed) is the production of spurious results (Kriegeskorte et al., 2010). Next to issues regarding ROI selection and analysis, defaults in specific analytical software can impact analysis outcomes. For instance, by default, SPM sequentially orthogonalizes all parametric modulators within the same model, which leads to potential effects of the order in which variables are entered. These are relevant in the type of choice models discussed here where multiple parametric modulators can represent different decision inputs (here healthiness and tastiness of food). We thus explore effects of variable order in an additional analysis branch. Finally, we base an analysis branch on novel findings obtained in our more highly powered replication sample.

## 2 MATERIALS AND METHODS

### 2.1 Study design

We conducted a preregistered (https://osf.io/qzyxm) direct replication of the data collection and analysis procedures described by Hare et al. (2009). Although we replicated all procedures as closely as possible, a few deviations from the original procedure were necessary to preserve the intended experience for study participants. These included:

1. Differences in the cultural context of the original study and this replication led to preregistered adjustments of stimuli. Details are described below.
2. The current study included additional measurement instruments that were not collected in the original study. All additional measures were collected after those required for replication in order to avoid biasing key responses. Additional measures are beyond the scope of the current manuscript.
3. The original study analyzed the data using SPM5, whereas we relied on SPM12.
4. Preprocessing of neuroimaging data was performed using SPM in the original study and fMRIPrep in this replication study.

Uncertainties in the interpretation of the original publication were resolved through e‐mail conversations with the lead author of the original study. Additionally, exploratory analyses were performed to further investigate potential causes of nonreplication of original study findings.

All procedures were approved by the ASCoR ethics committee of the University of Amsterdam (registered under: 2018‐PC‐9107) and all participants provided fully informed consent.

Deviations from preregistration include the above points 3 and 4. Personal communication with the authors (after preregistration) yielded a few small discrepancies between the methods described in the supplemental materials of the original study and the actual conduct for the inclusion criteria and general linear model (GLMs). For these we deviate from the preregistration but the procedures we employed here are equal to the original study. These deviations from the preregistration are clearly described in the respective methods sections. Further, in the preregistration, we mentioned we would add several analysis branches to the direct replication of the originally reported analysis pipelines to assess the robustness of the findings but these analyses were not described in detail yet. These exploratory (nonpreregistered) analyses are clearly marked in the results section.

### 2.2 Participants

Here, 80 participants (40 females, mean age 24.94 years; age range 18–43 years) completed this experiment, which was conducted in the Netherlands. After adjusting the group definition criteria (see details below), 15 participants qualified as SC (group) based on their food choices during the scanner task (9 female, mean age = 25.4, range = 20–34, mean BMI = 22.22, SD = 2.50) and 65 for as NSCs group (31 female, mean age = 24.83, range = 18–43, mean BMI = 22.89, SD = 2.64). SC and NSC groups did not differ significantly in gender distribution (*χ*(1) = 0.328, *p* = .567), age (*t*(78) = −0.399, *p* = .691) or BMI (*t*(77) = 0.897, *p* = .373). A power analysis, employing the fMRI power package (Mumford & Nichols, 2008), can be found in the preregistration.

#### 2.2.1 Recruitment strategy

Similar to the original study, participants were recruited at local gyms and sportclubs, through online sports and fitness groups, and on campus. Participants received 50 euro as reimbursement. This is a similar amount as in the original study.

#### 2.2.2 Inclusion criteria

An online screening survey was used to assess eligibility for the study. Participants were eligible for the study if they self‐reported (like participants in the original study) that “they enjoyed eating sweets, chocolate, and other ‘junk food’ even though they might be restricting them from their current diet” (Hare et al., 2009, supplemental materials). We also replicated the other (self‐reported) inclusion criteria from the lab manual of the original study: being between 18 and 45 years old, being right‐handed, being healthy, not having a history of psychiatric, neurological of metabolic illness, not using medication that interferes with fMRI, not being allergic to any of the foods used as stimuli, not being vegan, not being claustrophobic, not having any unremovable metal in the body.

In addition to the (preregistered) inclusion criteria reported in the original study manuscript, two additional criteria were implemented based on personal communication with the original study team. First, participants in the original study all indicated they are currently dieting to reduce their weight or to maintain their current weight.^2^ All participants in our replication sample fulfilled this criterion. Second, participants had either successfully lost weight in the past month or lost weight in the past and have maintained this lower weight in the past month. Six out of 80 participants in our replication sample fulfilled this criterion as well.

#### 2.2.3 Participant classification

Study participants were categorized as successful SC (the SC group) and unsuccessful SC (the NSC group) based on their choices in the fMRI task. In the original procedure, Hare et al. (2009) applied three criteria: (1) participants exercised self‐control in more than 50% of trials that required it (declining Liked‐Unhealthy items or choosing Disliked‐Healthy items); (2) in a multiple regression model estimated regressing food decisions on health and taste ratings for each individual participant, the effect of health ratings was larger than that of taste ratings; and (3) the *R* ^2^ of a simple regression fit to data from individual participants, regressing food decisions on health ratings is larger than the *R* ^2^ of a simple regression, regressing food decisions on taste ratings. Participants who met all three criteria were classified as the SC group and those who did not meet any of the criteria as the NSC group. In our sample, only 6 out of 80 participants qualified as successful SC according to these criteria and 8 participants did not classify as either SC or NSC group. In order to guarantee sufficient power for analyses requiring group comparisons, we preregistered the deviation from this procedure by basing the subject classification exclusively on a minimally relaxed version of one of the three criteria described by Hare et al. (2009), namely: Participants were classified as SC if they successfully executed self‐control on at least (compared to “more than” in the original study) 50% of trials that required self‐control (i.e., decline liked‐unhealthy items or choose disliked‐healthy items). As shown in Figure 3, the SC group defined based on this single criterion showed behavioral responses to the stimuli that are comparable to those reported in the original publication. For this analysis, Strong No and No responses were counted as a “no,” and Strong yes and yes responses were counted as a “Yes” (Hare et al., 2009, SI, p. 2).

### 2.3 fMRI task and stimuli

#### 2.3.1 fMRI task

The fMRI paradigm consisted of three parts, namely a health rating block, a taste‐rating block and a decision block and fully replicated the design of the original study. All subjects started with the rating blocks. The order of the rating blocks was counterbalanced: half of the participants first completed the health block, the other half started with the taste block. In each block, a total of 50 different food items was shown, including junk foods (e.g., chips or candy bars) as well as healthy snacks (e.g., apples or carrots). In the health rating block, participants were instructed to indicate the healthiness of the foods regardless of their taste on a 5‐point scale with as options: Very unhealthy—Unhealthy—Neutral—Healthy—Very healthy.

In the taste‐rating block, participants were instructed to indicate the taste of the foods regardless of their healthiness on a 5‐point rating scale with options: Very bad—Bad—Neutral—Good—Very good. The scale was shown below each food item. During each trial, the food stimulus and rating scale were presented for a maximum of 4 s and participants could indicate their rating with a button press. Immediately after responding, a feedback screen (0.5 s) was shown indicating their response, followed by a random intertrial interval with a duration uniformly distributed between 4 and 15 s.

Subsequently, participants were shown a reference food which they had rated neutral on both taste and health. If such a reference did not exist, a stimulus neutral on taste and healthy on the health scale was presented as a reference. Prior to the decision block, participants were instructed that on each trial they would have to choose between eating the food item shown in that trial or the reference food. They were also instructed that they would be required to eat the food they chose in a randomly selected trial at the end of the study session. Subjects were asked to indicate the strength of their preference on a 5‐point scale with as option: Strong no (=choose reference)—No (=choose reference)—Neutral—Yes (=choose shown item)—Strong yes (=choose shown item). The decision ratings are also referred to as the goal value of the food item. Equal as in the rating blocks, during each trial the food stimulus and rating scale were presented for a maximum of 4 s and participants could indicate their rating with a button press. Immediately after responding, a feedback screen (0.5 s) was shown indicating their response, followed by a random intertrial interval with a duration uniformly distributed between 4 and 15 s. For all three parts of the fMRI paradigm, the mapping of the response options (left–right) was counterbalanced across subjects.

**FIGURE 1:**
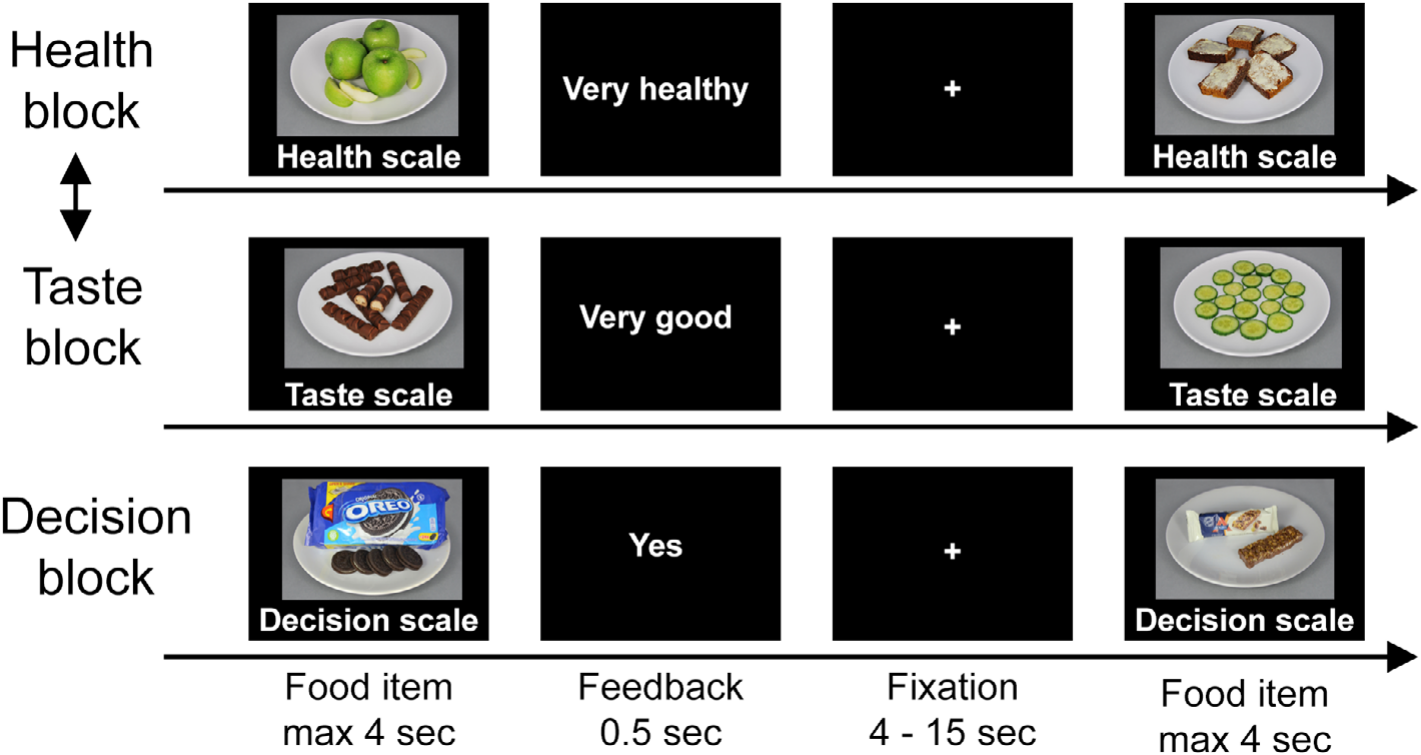
Task trial structure

The task trial structure is depicted in Figure 1.

#### 2.3.2 Stimuli

**FIGURE 2:**
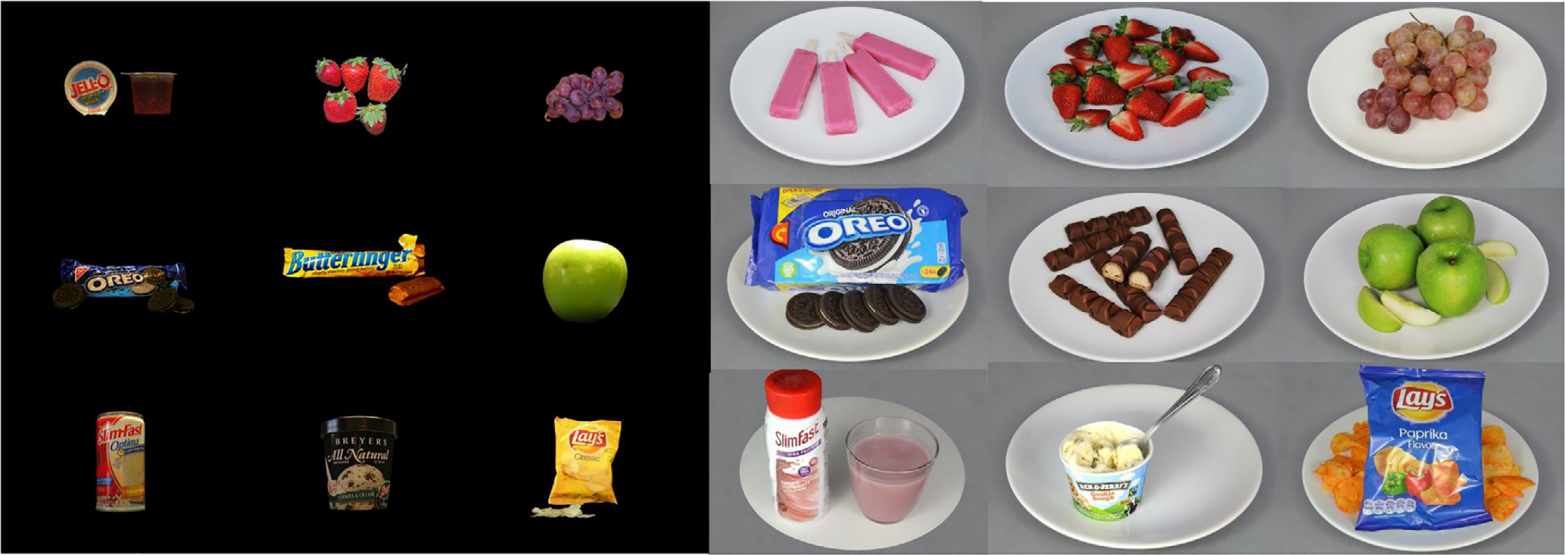
Selected stimuli or the original study (left panel) and the replication study (right panel). Stimuli from original study reproduced with permission from the original authors

Because this replication was conducted more than a decade after the original study several brands and products no longer existed. Further, the original study was conducted in a different cultural context and several of the original (U.S. American) stimuli are not sold or typically eaten in the Netherlands, the country where this replication was conducted. For these reasons, we developed a new set of stimuli for the replication study. To elicit similar value and self‐control related responses as in the original study it is important that the replication stimulus set has a similar mean and variation in health and taste ratings as the original set. Similar to the original study, the replication set consisted of junk foods as well as healthy snacks, bland foods, and dieting products; and included both packaged and unpackaged foods. We selected images from the validated F4H image set (Charbonnier et al., 2016). These stimuli were supplemented with 10 newly created stimuli including packaged foods and dieting products to represent the full range of products presented in the original study. The stimuli were pretested in an online and a lab pilot study (with exactly the same design as the fMRI study but no fMRI measurements were taken). These pilot studies showed that the mean level of healthiness and tastiness of the replication study's stimuli was similar to that of the original study. In the replication fMRI study, the mean healthiness rating was −0.35 (SD = 1.41) and the mean tastiness rating was 0.57 (SD = 1.10).^3^ For comparison, in the original study, the mean healthiness rating was −0.29 (SD = 1.29) and the mean tastiness rating was 0.57 (SD = 0.52). Differences between the two stimulus sets in terms of health ratings (95%CI[−0.606, 0.466]; *t*(98) = 0.259, *p* = .796) and taste ratings (95%CI[−0.342, 0.342]; *t*(98) = 0, *p* = 1) were not significant. Furthermore, the proportion of trials requiring self‐control, liked healthy, unliked healthy, liked unhealthy and unliked unhealthy trials was highly similar as well. Please see Tables S1 and S2 for a more complete description of the health and taste ratings of the stimuli in the pilot studies. Figure 2 shows example stimuli from the original study and the replication.

**FIGURE 3:**
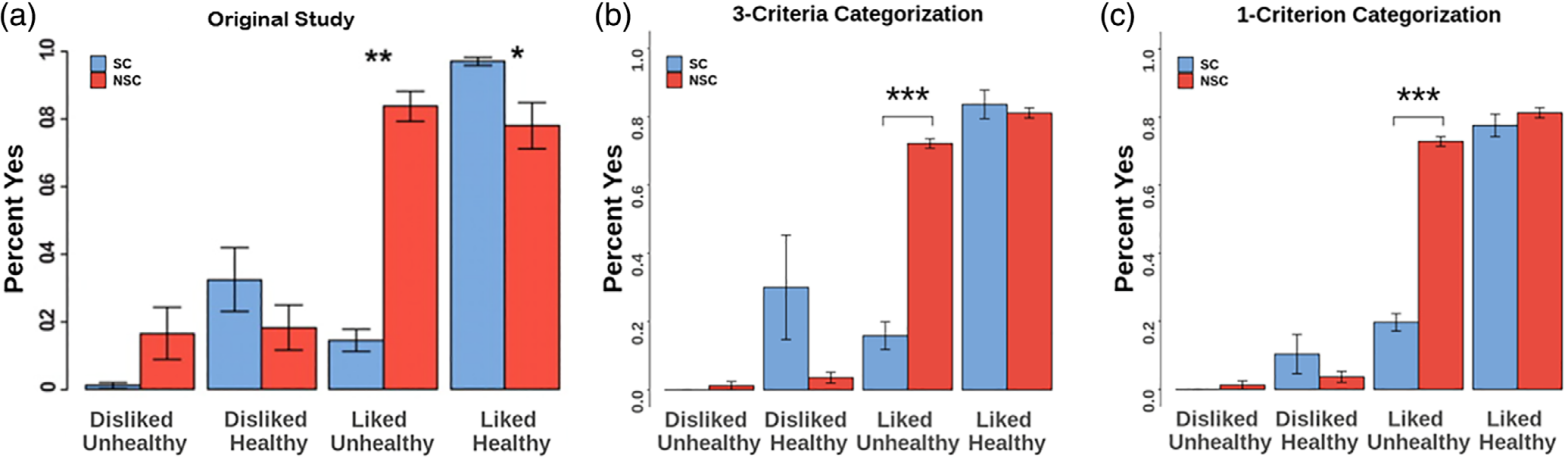
Proportion of “yes” and “strong yes” answers per type of food compared to the reference item for self‐controllers (SC) and non‐self‐controllers (NSC) (a) in the original study from Hare et al. (2009), reprinted with permission from AAAS, (b) in the replication sample applying the full set of categorization criteria, and (c) in the replication sample applying only one classification criterion. Error bars denote standard errors.

### 2.4 Procedures

After inclusion, participants were invited to the lab for a scan session. Participants were asked to refrain from eating for 3 h before the scan session. After arriving in the lab, participants were instructed about the study procedures and they completed informed consent. Thereafter, participants were scanned with a 3 Tesla Philips Achieva scanner (Philips Healthcare, Best, Netherlands). First, a T1‐scan was collected, followed by functional runs during which the fMRI task was carried out. Finally, resting‐state data were collected (these data are reported elsewhere; Speer et al., 2022). After leaving the scanner, participants completed a questionnaire on trait self‐control (Tangney et al., 2004), reward sensitivity (BIS/BAS), the occurrence of food in social interactions, smoking status, use of (hormonal) contraceptives, educational level, and they performed a go‐no‐go task. In the week after the experiment, the perceived temptation strength of the personal food environment was measured with EMA. The questionnaires and task completed after the scans and the EMA data are beyond the scope of the current manuscript.

### 2.5 fMRI data acquisition

A T1‐weighted structural scan was acquired for each participant (TR = 8.2 ms, TE = 3.9 ms, flip angle = 8°, slices = 220, voxel size = 1 × 1 × 1 mm, total scan duration = 363 s). A T2*‐weighted echoplanar imaging (EPI) sequence was used for the functional scans (TR = 2000 ms, TE = 27.63 ms, flip angle = 76.1°, slices = 36, voxel size = 3 × 3 × 3 mm, volumes = 343 per run, interleaved acquisition, field of view = 240 × 240 mm).

### 2.6 fMRI data analysis

As with all other procedures, our fMRI data analysis pipeline mirrored the procedures described in the original study as closely as possible.

#### 2.6.1 Preprocessing

Results included in this manuscript come from preprocessing performed using *fMRIPprep* 1.1.7 (Esteban, Markiewicz, et al., 2018, Esteban, Blair, et al., 2018; RRID:SCR_016216) with standard options, which is based on *Nipype* 1.1.3 (Gorgolewski et al., 2011, 2017; RRID:SCR_002502). Specifically, both preprocessing pipelines included correction for slicetime acquisition, motion correction, spatial normalization to the Montreal Neurological Institute EPI template, smoothing with an 8 mm FWHM Gaussian kernel, intensity normalization, and high‐pass temporal filtering (using a filter with 128 s cut‐off).

#### 2.6.2 General linear models

We used SPM12 to fit first‐and second‐level models to the MRI data. All whole‐brain maps and tables are thresholded in accordance with the original study at *p* < .001 and/or *p* < .005 (uncorrected). In addition, we report maps corrected for multiple comparisons at FDR *p* < .05.

##### First‐level models

For each participant, we estimated five GLMs with AR(1) to replicate the analyses presented in the original study. Table 1 presents a list of regressors included in each first‐level GLM as well as the task period which is modeled by the respective GLM. All feedback durations were not modeled and thus included in the baseline rest period, following procedures applied in the original study. All boxcar regressors were modeled with a duration equal to the participants' reaction time for that trial. All models further included six motion parameters and session constants as regressors of no interest.

**TABLE 1:** Task period and key regressors per GLM (note that all models further included six motion parameters and session constants as regressors of no interest)

GLM	Task period	Regressors
1	All trials	Three boxcar regressors indicating, respectively: Health trials Taste trials Decision trials Three regressors representing the product of each of the three boxcar regressors named above and a parametric modulator of goal value (i.e., decision trial ratings for each food item). A boxcar regressor indicating trials with missing goal value ratings and/or missing taste/health ratings, respectively.
2	Decision trials	Six boxcar regressors indicating: “Strong Yes” decisions “Yes” decisions “Neutral” decisions “No” decisions “Strong No” decisions Trials with missing goal value ratings.
3a	All trials	Three boxcar regressors indicating: Health trials Taste trials Decision trials Six regressors representing the product of each of the three boxcar regressors named above with each of two parametric modulators: Health rating Taste rating A boxcar regressor indicating trials presenting food items with missing health or taste ratings. In this model, the parametric modulator “health rating” was entered first.
3b	All trials	Model 3b is identical to Model 3a, except for the order of parametric modulators. In Model 3b, the parametric modulator “taste rating” was entered first.
4	Decision trials	Four boxcar regressors indicating: Trials in which self‐control was successfully employed Trials in which self‐control was not required Trials in which a neutral response was given Trials depicting food items for which any of the three ratings (health, taste, decision) was not available, since all ratings were necessary to categorize trials as described above.
5	Decision trials	Five boxcar regressors: Trials presenting liked, unhealthy items Trials presenting disliked, healthy items Trials which did not require self‐control Trials in which a neutral response was given Trials depicting food items for which any of the three ratings (health, taste, decision) was not available, since all ratings were necessary to categorize trials as described above

We estimated two versions of GLM3 as a methodologically driven branch extending the replication analyses in order to estimate order effects with regards to the two parametric modulators included in the model. Note that GLM5 was not preregistered or described in the original manuscript. We inferred its structure based on descriptions in the original manuscript (Hare, Science, 2009), which could not have been created based on GLMs 1–4.

##### Second‐level models

To fully replicate the analyses reported in the original study, we estimated second‐level models based on GLM1 and GLM4 (see Table 1). Specifically, for GLM1, we estimated a second‐level one‐sample *t* test including all subjects using SPM12. For GLM4, we estimated a two‐sample *t* test on successful self‐control trials comparing the SC to the NSC group using SPM12. All whole‐brain maps are presented at two thresholds, following the original publication: *p* < .001, uncorrected, and *p* < .005 uncorrected. No second‐level models were estimated for GLM2, 3, and 5 as these models were not needed to replicate the analyses presented in the original manuscript or to answer the research questions.

#### 2.6.3 PPI analysis

We replicated the PPI analysis based on the procedure described in Hare et al. (2009), using the SPM software (version 12). To extract left dlPFC activity during self‐control, we computed individual average time‐series within a 4 mm sphere surrounding individual subject peaks within a meta‐analytically defined seed region involved in self‐control in left dlPFC (see Figure S2 for details). Variance associated with the six motion regressors was removed from the extracted time‐series. The location of the peak voxels was based on a GLM specifically designed for this PPI analysis and modeled based on descriptions in the original study. As per the original procedure, this GLM was estimated on decision block trials only and included two regressors: one boxcar function identifying trials where a healthy food was shown, and one identifying trials in which an unhealthy food was presented, with motion parameters as regressors of no interest. Individual subject peaks within the left dlPFC self‐control mask were then identified based on voxels with the strongest positive response in unhealthy foods in that GLM. The seed time‐courses were then deconvolved in order to construct a time series of neural activity at that region.

For the first PPI analysis (PPI1), a second GLM (again estimated on decision block trials only) had the following regressors: (a) an interaction between the neural activity in the seed region and an indicator function for unhealthy trials; (b) an indicator function for unhealthy trials; and (c) the neural activity in the seed region. The first two regressors were convolved with a canonical form of the hemodynamic response. The model also included motion parameters as regressors of no interest.

For the second PPI analysis (PPI2), we identified a cluster in left IFG/BA46 in PPI1 as seed region. This analysis was identical to PPI1, except that the time‐series were extracted from that seed region.

## 3 RESULTS

### 3.1 Classification into SC and NSC

Although we applied identical recruitment criteria and methodology, the proportion of SC, given the original three‐criteria definition was much lower in the replication (SCs, *N* = 6; NSCs, *N* = 66) compared to the original sample (SCs *N* = 19; NSCs *N* = 18). To guarantee sufficient power to determine between‐group effects, we report results based on a preregistered less stringent definition of SC (*N* = 15) and NSC (*N* = 65) participants (see Section 2 for details).

Under this one‐criterion definition (applying self‐control in at least 50% of trials that require it), the SC and NSC participants in our replication study behaved substantially similar compared to participants in the original study as well as compared to replication participants grouped according to the full three‐criteria definition used in the original study (see Figure 3). In both one‐ and three‐criteria group definitions, the proportion of "Yes" and "Strong Yes" answers to liked unhealthy items was much lower in SC participants (*M*_3‐Criteria_ = 0.16; *M*_1‐Criterion_ = 0.19) compared to NSC participants (*M*_3‐Criteria_ = 0.72; *t*(70) = 6.28, *p* < .0001; *M*_1‐Criterion_ = 0.73, *t*(78) = 9.59, *p* < .0001), replicating original study results. In other words, SC participants exhibited more self‐control than NSC participants. Further, similar to the original study, group differences in responses to disliked, unhealthy and disliked, healthy food items were not significant. Overall, choice probabilities are similar across both one‐ and three‐criteria group definitions in the replication sample and across the original and replication samples. We were not able to replicate differential group responses to liked, healthy items in the one‐ or three‐criteria group definitions.

Further replicating the original findings, SC and NSC participants did not differ significantly in their mean health ratings (*t*(78) = 0.469, *p* = .640). In an additional analysis, extending the original analysis pipeline, we further found very high correlations between average ratings per stimulus between groups for both health (*r* = .989, 95%CI[0.981–0.994], *t*(48) = 47.31, *p* < .0001) and taste (*r* = .913, 95%CI[0.852, 0.950], *t*(48) = 15.55, *p* < .0001) ratings. Together, these two analyses indicate that the groups showed similar awareness of the foods' healthiness and had similar perceptions of the stimulus set (see supplementary Table S3 for group‐wise reaction time data). Overall, the behavioral data show that the responses of the replication sample to the scanner task were substantially similar to those in the original sample despite the novel stimulus set, different cultural context, and overall lower proportion of participants classified as SC.

### 3.2 Gender

Before testing the main hypotheses, we followed procedures outlined in the original study to examine gender effects as a potential confound in the main analyses. Similar to findings reported in the original study, our SC group contained somewhat more females (60%) than males. We thus examined the role of gender in SC‐NSC differences. First, we replicated a regression analysis, relating the effect of stimulus health ratings on decision ratings of the same stimuli to the effect of health ratings on vmPFC activity in response to the same stimuli during decision trials (as estimated in GLM1), including gender as a predictor. Replicating results reported in the original study, the gender effect was not significant (*B* = 0.011, SE = 0.101, *t* = 0.113, *p* = .910) while the relationship between health and decision ratings remained significant (*B* = 0.636, SE = 0.221, *t* = 2.877, *p* = .005). Second, we found that gender was unrelated to the extent to which vmPFC activity during decision trials scaled with health (*t*(78) = −1.371, *p* = .174) and taste ratings (*t*(78) = 0.721, *p* = .473) as estimated in GLM3.

### 3.3 Hypothesis 1: Activity in vmPFC is correlated with participants' goal values regardless of whether or not they exercise self‐control

**FIGURE 4:**
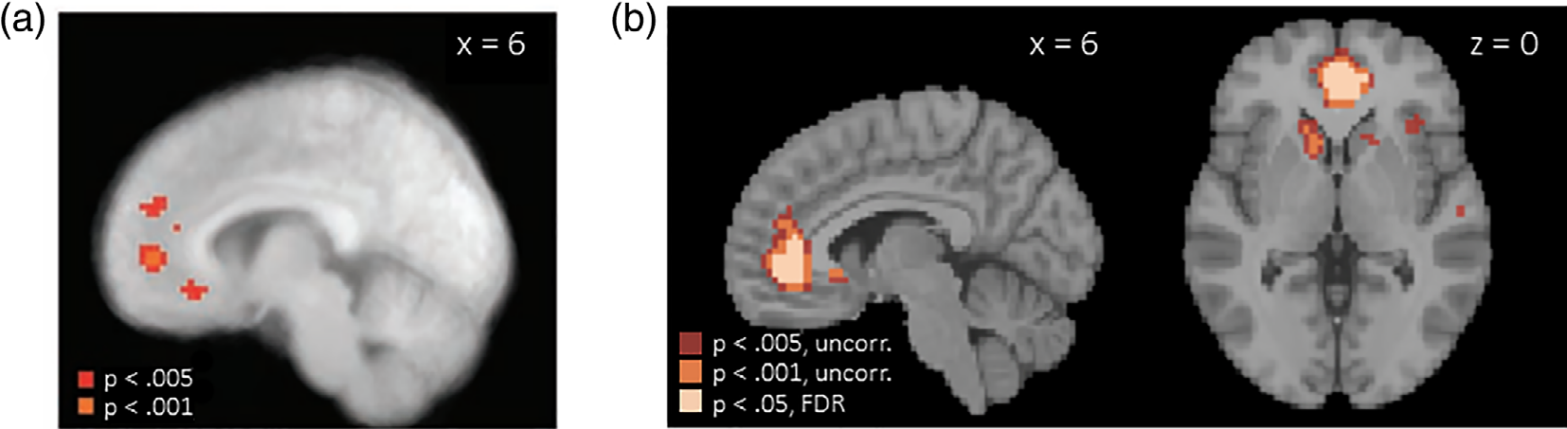
Representation of goal value in ventromedial prefrontal cortex (vmPFC) (a) in the sample collected by Hare et al. (2009); reprinted with permission from AAAS and (b) in the present sample. All maps in this figure are thresholded at *p* < .005 and *p* < .001, uncorrected. Additionally, panel (b) is thresholded at *p* < .05 FDR

Our data support the first hypothesis, largely replicating the original results. Specifically, interrogating GLM1, we found that brain activity within vmPFC was more strongly correlated with goal value (i.e., food choices self‐reported during the decision block of the scanner task) during decision compared to taste‐rating trials. Significant clusters within vmPFC in our sample were located in close proximity to those reported in the original study (Figure 4, Table S4). Activity extracted from the vmPFC cluster shown in Figure 4b (thresholded at *p* < .005, uncorrected) was further significantly associated with goal value in both the SC and NSC groups (see Figure S1). In contrast to the original study, we additionally found significant associations between goal value and brain activity in bilateral striatum in the same contrast. Extending the original analysis pipeline, Figure 4b further highlights voxels in which the relationship between brain activity and goal value remained significant under multiple comparison correction (FDR *p* < .05). Only voxels in vmPFC, but not striatum were significant at that threshold.

**FIGURE 5:**
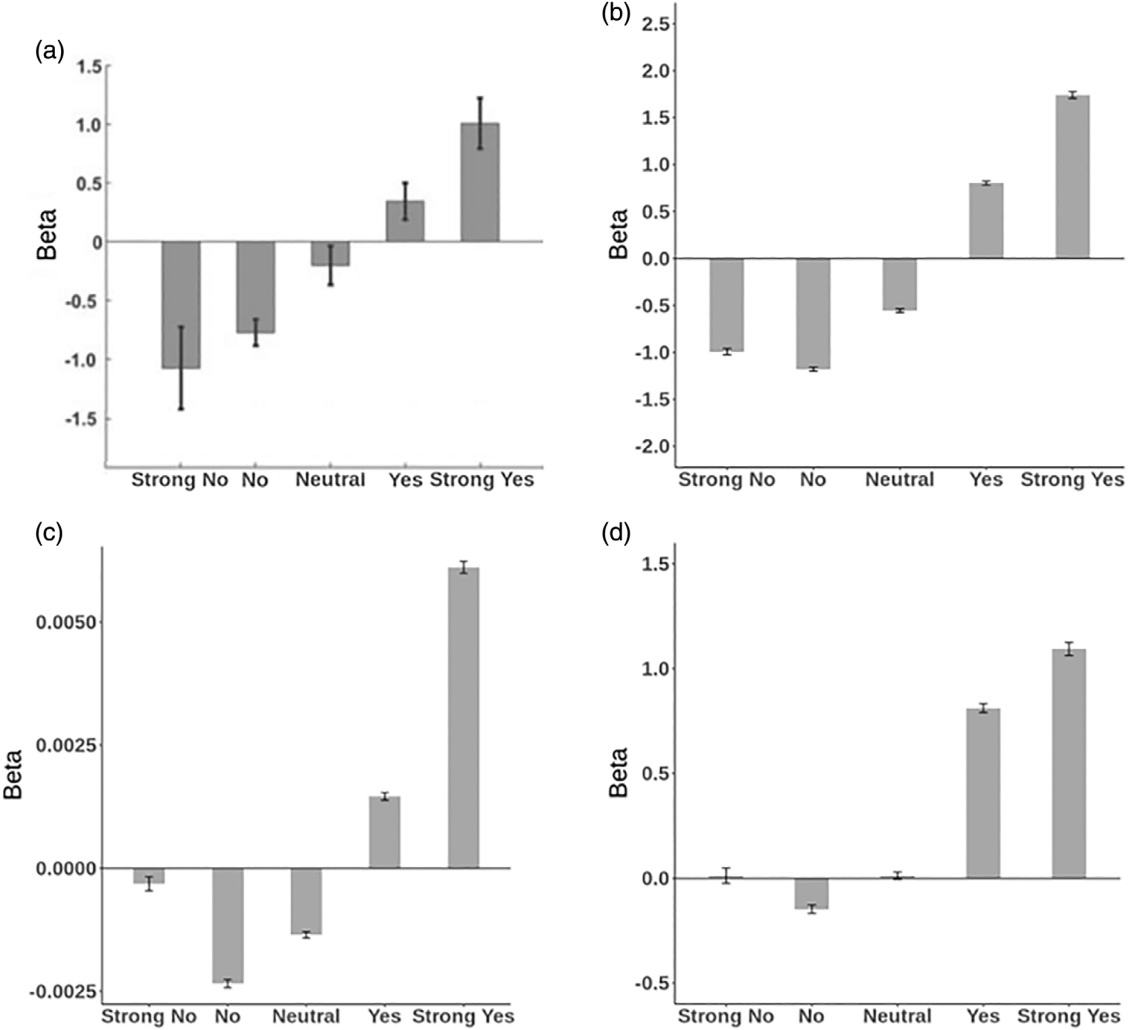
Encoding of goal‐value in ventromedial prefrontal cortex (vmPFC) and striatum. (a) Findings of the original study based on individual peak voxels within the region‐of‐interest (ROI) in Figure 4a, from Hare et al. (2009), reprinted with permission from AAAS, (b) findings derived from the present study based on individual peak voxels within the ROI in Figure 4b intersected with an anatomical vmPFC mask, (c) findings derived from the present study based on average beta values across voxels within the ROI in Figure 4b intersected with an anatomical vmPFC mask, and (d) findings derived from the present study‐based individual peak voxels within the ROI in Figure 4b intersected with an anatomical caudate mask

Further replicating the original study, GLM2 results showed a clear differentiation in brain activity between goal value levels (“Strong no”—“Strong yes” decisions) within peak voxels (individually identified per participant within the vmPFC cluster shown in Figure 4b, thresholded at *p* < .005, uncorrected (see Figure 5).

Note that the analysis decisions made in the original study and replicated here are optimized to produce the pattern shown in Figure 5a (the published result from the original study). The analysis focuses on a single voxel within a larger vmPFC area which shows the strongest positive correlation with goal value for each individual participant. In other words, these results show that, on average, study participants have at least one voxel in vmPFC that shows the pattern of interest. To further test the generalizability of this finding, we added an analysis branch not included in the original study where we examine whether this step‐wise representation of goal value is present more broadly across the entire vmPFC region that was found to correlate significantly (at *p* < .005, uncorrected) with goal value (see Figure 4b). Indeed, we found conceptually similar results after averaging voxel‐wise estimates across the vmPFC mask in Figure 4b for each study participant (Figure 5c). Of note, “Strong no” responses showed somewhat unexpected neural responses given this ROI definition and overall beta estimates were substantially smaller than in the original study (Figure 5a) and replication of the original analysis pipeline (Figure 5b), suggesting that more data are necessary to identify goal value representations within a broader vmPFC mask. This is important information for power calculations of follow‐up work relying on these findings for ROI definitions, because most follow‐up studies will be unable to include a goal value localizer task to identify individual‐level subject peak voxels.

In a second analysis branch extending the pipeline of the original study, we further examined the representation of goal value within the striatum clusters identified in Figure 4b. We applied the analysis approach of the original study and extracted data from individual subject peaks within the striatal clusters. These striatal clusters are the voxels within the mask of the GLM 1 whole‐brain map (thresholded at *p* < .005, uncorrected) that fall within the AAL bilateral caudate mask. Figure 5d supports the idea that the neural representation of goal value is not fully unique to vmPFC. Even in individual peak‐voxels within our caudate ROI, we see some evidence of differentiation between individual levels of goal value, at least when applying the optimized analysis procedure of identifying individual peak voxels. Table S5 provides means and standard deviations per goal value level for Figure 5b–d.

### 3.4 Hypothesis 2: Activity in the vmPFC should reflect the health ratings in the SC group but not in the NSC group

**FIGURE 6:**
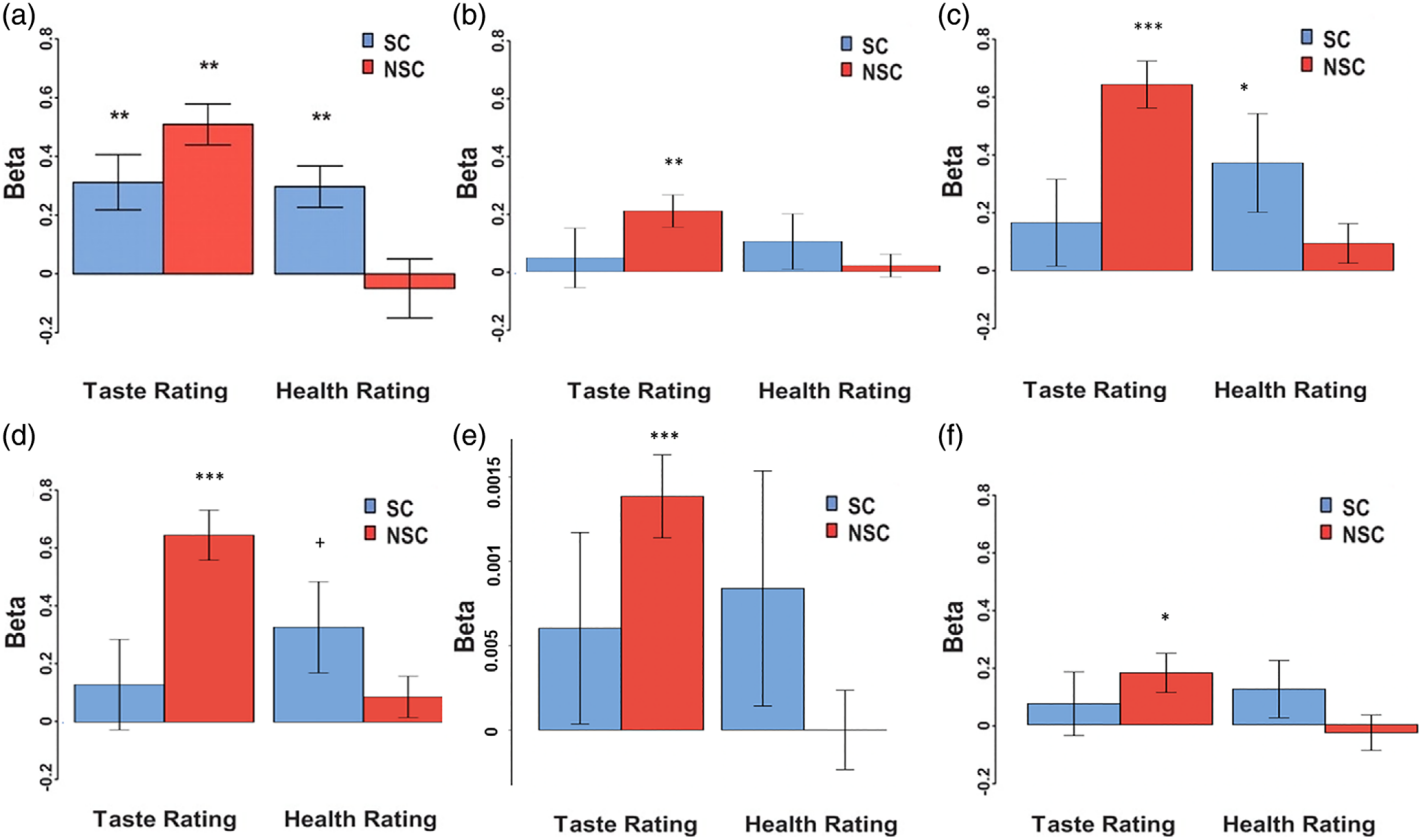
Relationship between ventromedial prefrontal cortex (vmPFC) activity and health/taste ratings in self‐controllers (SC) and non‐self‐controllers (NSC); (a) original study result reported in Hare et al. (2009) based on activity estimates extracted from individual peak voxels, reprinted with permission from AAAS and (b) replication study based on peak voxels within vmPFC with parametric modulators entered in the order: taste, health, including all trials. All of the following panels exclusively include decision trials: (c) replication result based on activity in peak voxels within vmPFC with parametric modulators entered in the order: taste, health; (d) replication result based on activity in peak voxels within vmPFC with parametric modulators entered in the order: health, taste; (e) replication result based on average activity across voxels within vmPFC; and (f) replication result based on activity in peak voxels within caudate; ***p < .001, **p < .005, *p < .05, +*p* = .06

Analyses based on our data replicated results presented in connection to Hypothesis 2 in the original study. First, please note that the original study did not explicitly limit this analysis to decision trials. However, we feel that this is the most logical choice from a theoretical standpoint and also find clearer replicated results in decision trials rather than when extending the analysis to all trials. For purposes of comprehensiveness and transparency, Figure 6b presents the results when including data from all trials in the analysis. However, we focus our discussion on results presented in Figure 6c–f, which exclusively include data from decision trials. In accordance with Hypothesis 2 and with findings reported in the original study, results based on GLM3 indicated that during decision trials SC participants showed significant encoding of health ratings in vmPFC (*M* = 0.369, 95% CI[0.007; 0.732], *t*(14) = 2.184, *p* = .046), whereas NSC participants did not show this effect (Figure 6c). In our data, there is no significant difference between the extent to which SC and NSC participants showed a relationship between vmPFC activity and health ratings (*M*_diff_ = 0.276 95%CI[−0.048; 0.599]; *t*(78) = 1.698, *p* = .093). This direct comparison (which is necessary to statistically establish a difference in the effects; Nieuwenhuis et al., 2011) was not reported in the original study.

Further, in line with the original study, NSC participants showed significant encoding of taste ratings (*M* = 0.638, 95% CI[0.477; 0.799], *t*(64) = 7.917, *p* < .0001), but, in contrast to the original publication, SC participants did not show this relationship in our replication study. Extending the original analysis pipeline, we found that these results are robust to the order with which parametric modulators were added to the model (Figure 6d). This is likely due to the fact that, like in the original study, health and taste ratings are not strongly correlated with each other (SC‐group: mean *r* = .032, NSC‐group mean *r* = .032). Additionally, we again examined the impact of the ROI analysis strategy, by averaging activity across all voxels within the vmPFC mask rather than identifying single voxel individual subject peaks (Figure 6e). Again, this analysis shows a similar pattern of results as the more optimized analysis pipeline which Figure 6a–d are based on, but the effect size is substantially smaller and the representation of health ratings in vmPFC among SC does not reach conventional levels of significance in this relatively large fMRI sample. Finally, an additional exploratory analysis showed a similar pattern of results when extracting activity from peak voxels in our caudate mask, but effect sizes were somewhat smaller than in vmPFC (Figure 6f).

**FIGURE 7:**
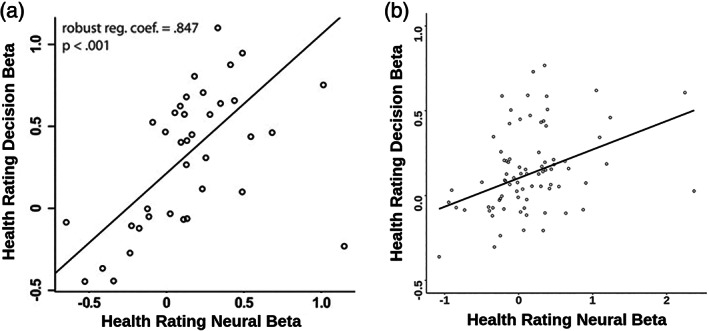
Relationship between encoding of health ratings in ventromedial prefrontal cortex (vmPFC) and effect of health ratings on decision‐making in the (a) original publication, Hare et al. (2009), reprinted with permission from AAAS, and (b) replication sample

Further in support of H2, we replicated the positive linear relationship between the effect of health ratings on decisions and the impact of health ratings on vmPFC activity as extracted from GLM3 (robust regression coefficient = 0.639, SE = 0.219, *p* = .005; Figure 7).

In sum, data from the replication study are in line with the idea that vmPFC activity represents health ratings, but group differences in this relationship are not significant in the replication sample and were not tested in the original study.

### 3.5 H3: The dlPFC should be more active during successful than failed self‐control trials

**FIGURE 8:**
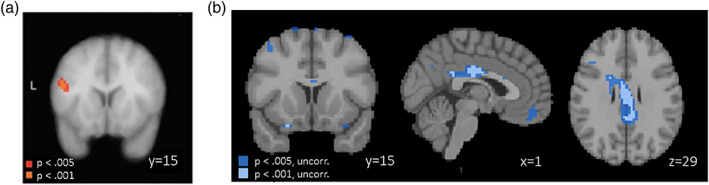
Self‐controllers > non‐self‐controllers (SC > NSC) during successful self‐control trials compared to rest in (a) the original publication (Betas in colored voxels are positive, meaning that activity is stronger during self‐control compared to rest.) footnote 1, from Hare et al. (2009), reprinted with permission from AAAS, and (b) in the replication sample (Betas in colored voxels are negative, meaning that activity is stronger during rest compared to self‐control.). All maps in this figure are thresholded at *p* < .005 and *p* < .001, uncorrected. Note that in panel (b), no voxels survive correction for FDR *p* < .05. This figure is based on GLM4. Footnote 1: The region identified in this analysis was labeled in the original paper as “dorsolateral prefrontal cortex,” but comparison of this activation location (MNI coordinates −48, 15, 24) shows that this region is much more likely to fall in the inferior frontal gyrus (which is generally not considered to fall within the dorsolateral region). However, for consistency with the original report, we will continue to use the dorsolateral prefrontal cortex (dlPFC) label

Our data are not consistent with the hypothesized role of dlPFC in self‐control. Specifically, when comparing SC and NSC participants during trials that require self‐control compared to baseline, only regions in which activity is negatively associated with this contrast emerge as statistically significant at the thresholds used in the original study (Figure 8b). Specifically, among others, clusters in (primarily left) dlPFC and vmPFC showed more negative activation during self‐control compared to rest. Of note, none of these clusters remained significant when applying more stringent correction for multiple comparisons. This suggests that behavioral self‐control in our sample was associated with a weaker subjective value response for a given food item as well as weaker dlPFC involvement rather than by enhanced dlPFC activity.

In the original publication, several follow‐up analyses focused on the cluster identified in the analysis presented in Figure 8a. A key motivation for these follow‐up analyses was to examine interactions between vmPFC and dlPFC as a potential mechanism of self‐control. Although we did not find evidence for the hypothesized role of average activity in dlPFC during self‐control, it is conceivable that this region still impacts self‐control decisions through functional connectivity with vmPFC. To allow us to test this hypothesis in the absence of a replication of the finding shown in Figure 8a, we deviated from the analysis pipeline reported in the original publication. Specifically, instead of further examining a dlPFC cluster retrieved from the analysis presented in Figure 8b, we created a new ROI mask which meta‐analytically represents processing associated with self‐control in dlPFC. Specifically, we intersected a mask taken from www.neurosynth.org with an anatomical mask of left dlPFC (see Figure S2). The anatomical dlPFC mask was based on the ROI definition from an earlier study (Gozzi et al., 2009): it was constructed by first combining the superior, middle, and inferior frontal gyri, and subsequently trimming the ROI at *x* > 10 or *X* < 10 and *z* > 1.

**FIGURE 9:**
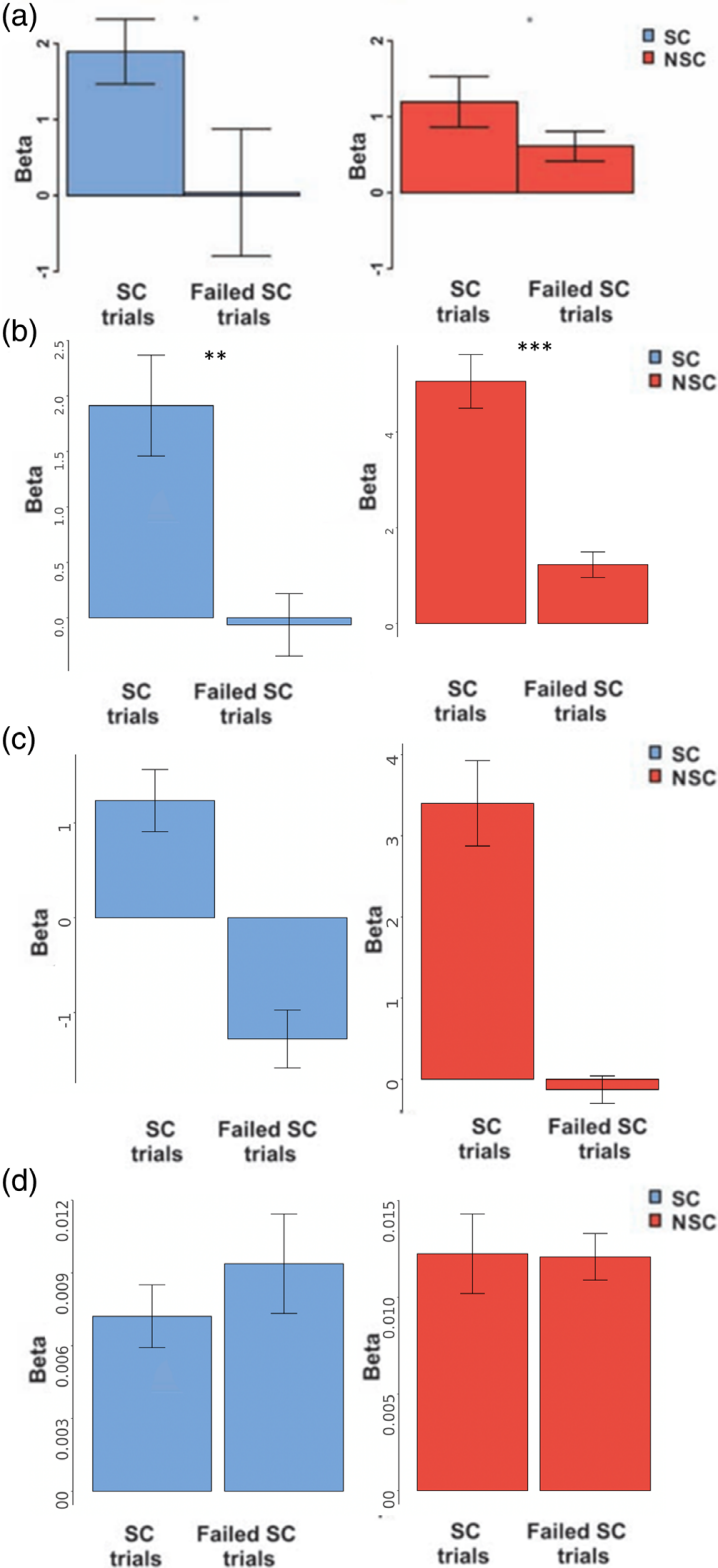
Brain activity in successful and unsuccessful self‐control trials (compared to rest) in self‐controllers (SC) and non‐self‐controllers (NSC), (a) activity extracted from individual subject peak voxels within a GLM 4 dorsolateral prefrontal cortex (dlPFC) region‐of‐interest (ROI) originally reported by Hare et al. (2009), reprinted with permission from AAAS, (b) activity extracted from individual subject peak voxels within a meta‐analytically defined dlPFC (“self‐control”) ROI in the current sample, (c) activity extracted from individual subject peak voxels within white matter in the current sample, and (d) average activity across a meta‐analytically defined dlPFC (“self‐control”) RO in the current sample

Using this intersected mask, we first followed the original analysis pipeline and identified individual subject peaks within the ROI for the contrast successful > unsuccessful trials which required self‐control as estimated in GLM4 and extracted signal from these peak voxels for the contrasts successful self‐control > rest and unsuccessful self‐control > rest. Even though Figure 8 shows a nonreplication with regards to average dlPFC activity during self‐control in a whole‐brain analysis, Figure 9b (Table S6) shows test results that replicate the original findings when examining activity in individually identified peak‐voxels within dlPFC. Using this analysis approach, we found greater dlPFC involvement in successful (vs. rest) compared to unsuccessful (vs. rest) self‐control trials in both SC and NSC participants.

As highlighted above, the replicated analysis pipeline is optimized to show that, on average, participants have at least one voxel in a general dlPFC area that shows a difference between successful and unsuccessful self‐control trials. Is the replicated finding in Figure 9b purely a result of the analysis strategy or meaningful evidence for the role of dlPFC in self‐control? To answer this question, we replicated the same analysis procedure, using a ROI that should not be differentially involved in successful and unsuccessful self‐control, namely white matter voxels. As shown in Figure 9c, when extracting signal from individual peak voxels in white matter, we seemingly produce evidence suggesting that white matter voxels are differentially involved in successful (vs. rest) compared to unsuccessful (vs. rest) self‐control trials in both SC and NSC participants.

In a final test examining the potential role of average dlPFC activity in self‐control processes in our sample with more stringent statistical methods, we implemented an additional analysis branch that was not included in the original study. Specifically, we extracted average activation across our meta‐analytically defined left dlPFC ROI rather than choosing the peak voxel per subject (see Figure 9c). This analysis demonstrates that, in our sample, activity within a left dlPFC ROI that is meta‐analytically associated with the concept of self‐control is not differentially involved in successful and unsuccessful self‐control trials.

### 3.6 H4: dlPFC and vmPFC should exhibit functional connectivity during self‐control trials

**FIGURE 10:**
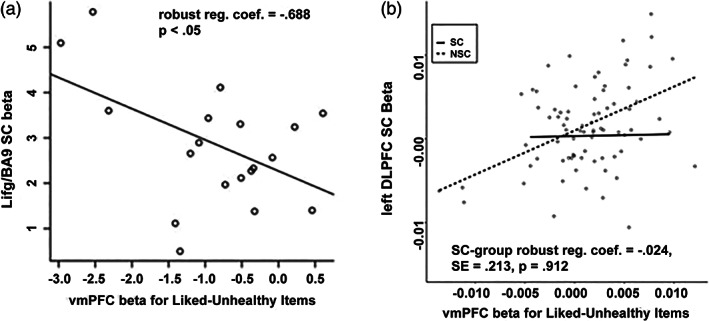
(a) Correlation between activity in left dorsolateral prefrontal cortex (dlPFC) during self‐control trials and ventromedial prefrontal cortex (vmPFC) activity during liked‐but‐unhealthy food item presentation in the self‐controllers (SC) group in the original study by Hare et al. (2009) reprinted with permission from AAAS and (b) regression of activity in meta‐analytic dlPFC region‐of‐interest (ROI) during self‐control trials on interaction of self‐control group (non‐self‐controllers [NSC] > SC) and vmPFC activity during liked‐but‐unhealthy food item presentation. In both panels, the printed robust regression coefficient is the main effect of vmPFC activity for the SC group

Does dlPFC play a role in self‐control through its functional connection with vmPFC rather than through average activation? This replication study produced mixed evidence with regards to H4. The first replicated test of H4 required a linear regression of left dlPFC activity during self‐control trials on activity within vmPFC in response to liked, unhealthy food items. This analysis is based on GLM5. Using our meta‐analytic left dlPFC mask, we do not replicate the negative relationship between dlPFC and vmPFC activity that was reported in the original study among SC participants (robust reg. coef. = −0.024, SE = 0.213, *p* = .912). We do see a significant interaction effect so that the dlPFC–vmPFC relationship is more positive in the NSC than the SC group (robust reg. coef. = 0.544, SE = 0.254, *p* = .035, Figure 10).

The final analysis in the original manuscript tested whether left dlPFC and vmPFC exhibited task‐related functional connectivity. Again, we relied on our meta‐analytically defined left dlPFC mask for this analysis. As in the original analysis, individual peak voxel for activity in response to unhealthy food within left dlPFC were used as seeds in the PPI analysis. Maps were thresholded at *p* < .005 uncorrected, following the original publication. In the original study, the authors expected to find a direct, negative connection between left dlPFC and vmPFC activity, but the data did not support that hypothesis. Instead, the authors reported a negative interaction between left dlPFC and IFG/BA46 (PP1) and, in a second step, a positive interaction between IFG/BA46 and vmPFC (PPI2). Of note, the whole brain table for PPI in the supplementary materials of the original publication (original study Table S4) does highlight a cluster in medial frontal gyrus (MNI coordinates 12, 51, 3) in which activity positively correlated with left dlPFC activity, contrary to expectations. This coordinate overlaps with the vmPFC region that was sensitive to goal value in our replication sample (Figure 4). In other words, in the original study, there was some unexpected evidence of positive connectivity between dlPFC and vmPFC activity.

**FIGURE 11:**
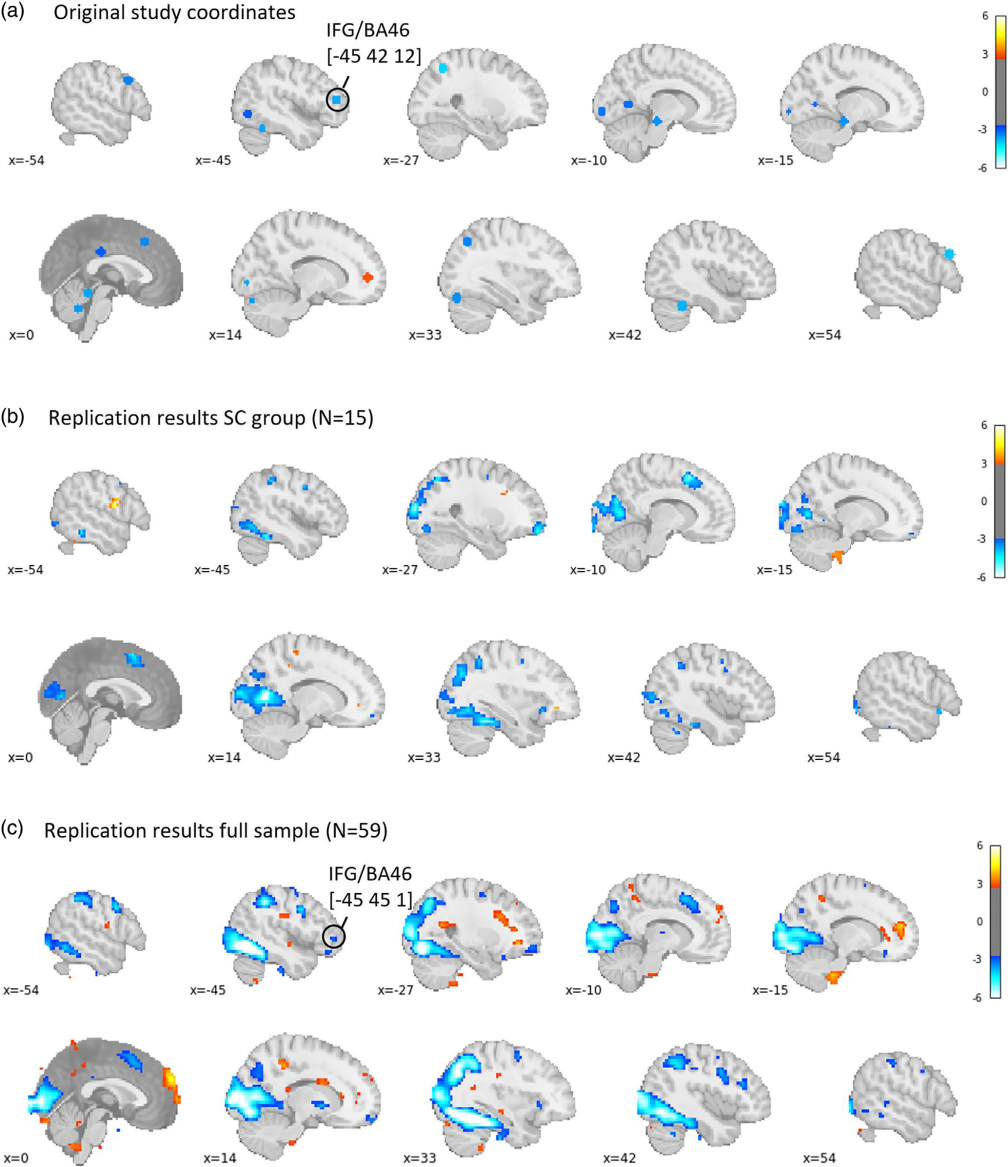
(a) Coordinates reported in the original study (Table S4 in the original manuscript; thresholded at *p* < .005 uncorrected; represented by 6‐mm‐radius spheres) showing task‐related functional connectivity with the left IFG/BA9 (−48 15 24) among self‐controllers (SC) group; (b) regions showing task‐related functional connectivity with peak voxels within custom left dorsolateral prefrontal cortex (dlPFC) mask among SC group in current study (*N* = 15), thresholded at *p* < .005 uncorrected; and (c) regions showing task‐related functional connectivity with peak voxels within custom left dlPFC mask among all participants in current study (*N* = 59), thresholded at *p* < .005 uncorrected

Replicating the original analysis pipeline, but deviating on the choice of left dlPFC ROI, we did not replicate the finding of negative functional connectivity between the left dlPFC seed voxels and left IFG/BA46 in PPI1 (Figure 11, Table S7). This could be evidence of nonreplication or a lack of statistical power, given the limited sample size of our SC group (*N* = 15). To create optimal conditions for replication, we again deviated from the original analysis and included all participants who exhibited any self‐control during the scanner task (*N* = 59) in the PPI analysis.

Within this sample, we found a small cluster of vmPFC voxels (peak [−15 33 –18], *k* = 12 voxels) in which activity was negatively correlated with activity in left dlPFC. In addition, consistent with the original findings, we found a small cluster around the left IFG/BA46 region ([−45 45 1], *k* = 10) in which activity was negative correlated with activity in left dlPFC.

**FIGURE 12:**
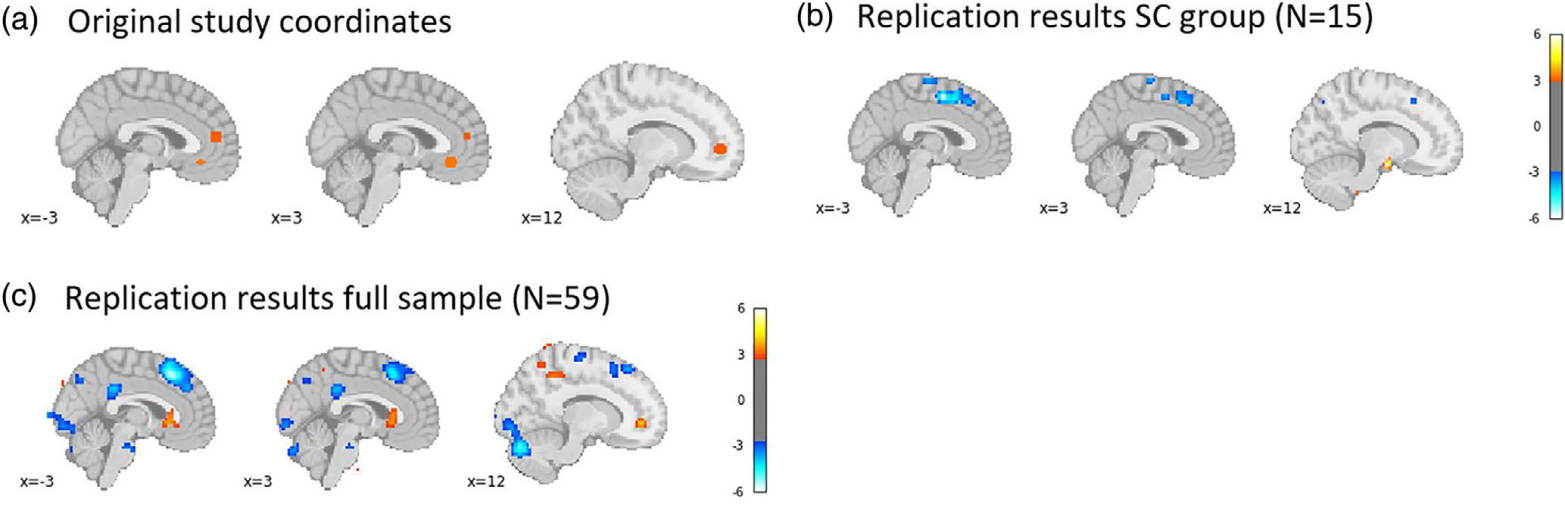
(a) Positive coordinates reported in the original study (Table S5 in the original manuscript; thresholded at *p* < .005 uncorrected; represented by 6‐mm‐radius spheres) showing task‐related functional connectivity with the left IFG/BA46 region (−45 42 12) among the self‐controllers (SC) group; (b) regions showing task‐related functional connectivity with the left IFG/BA46 region (−45 45 1) among the SC group in current study (*N* = 15), thresholded at *p* < .005 uncorrected; and (c) regions showing task‐related functional connectivity with peak voxels the left IFG/BA46 region (−45 45 1) among all participants in the current study (*N* = 59), thresholded at *p* < .005 uncorrected

Given the replication of PPI1 findings in left IFG/BA46 for the full sample of participants who exhibited any self‐control, we proceeded with PPI2, using the new left IFG/BA46 seed ([−45 45 1]) obtained in PPI1 (Figure 12, Figure S4, Table S8). Applying the original analysis pipeline to only SC group participants (*N* = 15), we again did not replicate the original findings of negative associations with vmPFC activity. However, when including all 59 participants in PPI2, we found positive task‐related functional connectivity with a cluster (peak [0 18 –2], *k* = 114) in vmPFC, although this cluster was somewhat more dorsal than the one found in the original study.

In sum, we find evidence that a cluster in left dlPFC that is meta‐analytically associated with self‐control showed direct negative, functional connectivity with a vmPFC cluster and also indirect (through left IFG) negative functional connectivity with another vmPFC cluster. Both of these results are in line with Hypothesis 4, but only the latter was actually found by the original authors. Further, both results only emerged when a larger proportion of participants were included in the analysis, pointing to potential issues with statistical power and/or the fact that these results may not be specific to the SC group.

## 4 DISCUSSION

Hare et al. (2009) were among the first to provide empirical evidence on the neural correlates of self‐control. Since then, this seminal study has had profound impact on theory and empirical work across multiple fields, but it has never been directly replicated. We performed a preregistered, direct replication of this experiment with two goals: (1) to further strengthen the evidence base for self‐control theory and research, and (2) to test the robustness of the original results across analytical choices. The results of the four key hypothesis tests are summarized in Table 2.

**TABLE 2:** Hypothesis test overview

Hypothesis (quoted from Hare et al., 2009, p. 646)	Replication findings
[Activity] in vmPFC should be correlated with participants' goal values regardless of whether or not they exercise self‐control	Supported
2 [A]ctivity in the vmPFC should reflect the health ratings in the SC group but not in the NSC group.	Supported, with reservations
3 [T]he dlPFC should be more active during successful than failed self‐control trials.	Not supported
4 dlPFC and vmPFC should exhibit functional connectivity during self‐control trials.	Mixed evidence

Abbreviations: dlPFC, dorsolateral prefrontal cortex; NSC, non‐self‐controllers; SC, self‐controllers; vmPFC, ventromedial prefrontal cortex.

Our data provide further support for the now widely accepted notion that decisions are associated with a value signal in vmPFC, which integrates relevant choice attributes to inform a final decision (Hypotheses 1 and 2; Table 2). Specifically, like Hare et al. (2009), we found positive correlations between participants' goal values (choices for food items) and activity within vmPFC, regardless of whether participants exercised self‐control. We were also able to replicate findings which were reported in the original study in support of the idea that vmPFC prioritizes choice attributes that are consistent with each individual's subjective values. Specifically, as in the original study, activity in vmPFC was associated with the perceived healthiness of food items in participants who were relatively more successful at exercising self‐control in the experimental task but not in participants who were relatively less successful. However, we did not find evidence of significant differences between the two groups. Overall, these results are in line with a broader set of literature in neuroeconomics, which has described the role of vmPFC in valuation across diverse types of stimuli (e.g., money, consumer goods, etc., for a review see (Bartra et al., 2013)). The present study is the first to provide a direct replication of this effect in the context of food‐related decision‐making. Thus, this replication study increases the confidence in choice models of self‐control which describe self‐control as a value‐based choice (Berkman et al., 2017).

In addition to the replication of the originally reported analyses, we added several analysis branches to further test the robustness of these results. First, in a follow‐up analysis to the whole‐brain search for brain regions associated with goal value (Figure 4), Hare et al. (2009) highlight the fact that individual scale points (−2 –2) of goal value are neatly distinguished in a step‐wise pattern in their vmPFC ROI, suggesting that the ROI can be used to precisely distinguish and predict choices. However, the original analysis approach was optimized to demonstrate this effect and requires individual‐level choice data to identify individual peak‐voxels within a larger vmPFC ROI. In addition, this analysis supports the limited conclusion that, on average, most study participants show this step‐wise encoding of goal value in at least one voxel within a larger vmPFC area. We added an alternative analysis approach by averaging signal extracted from all voxels within the vmPFC ROI in which activity was associated with goal value in our replication sample. We show that the step‐wise encoding of choice behavior is largely preserved in this more general analysis, but that the effect size is substantially smaller. Similarly, when examining relationships between health and taste ratings and average signal within vmPFC, we do not find significant encoding of health ratings in the SC group despite the relatively large size of this replication sample. In other words, future studies that are interested in reusing these vmPFC ROIs as indicators of goal value without the luxury of an individual‐level localizer task that allows them to identify individual peak voxels per person likely require a much larger sample to be appropriately powered than implied by the original publication.

Further, next to vmPFC and in contrast to the original study, we identified positive associations between goal value and activity in clusters within the striatum at a relatively lenient statistical threshold (*p* < .001, uncorrected) used in the original study. This discovery is likely a function of the increased power in the larger replication sample and largely in line with the neuroeconomics literature on subjective valuation which regularly identifies clusters in both vmPFC and striatum (Bartra et al., 2013). Following up on this finding, we found some evidence of differentiation between individual levels of goal value, even within our caudate ROI when applying the optimized analysis procedure reported in the original study. This adds to the findings in prior work suggesting that vmPFC is not the exclusive locus of goal value representation in the human brain.

We did not find strong evidence in support of the second set of hypotheses (Hypotheses 3 and 4, Table 2) proposed by Hare et al. (2009), which highlight the role of left dlPFC in self‐control. First, we examined average activity levels in left dlPFC. Even though there were clear (and replicated) behavioral differences between participants who were relatively more and those who were relatively less successful at exercising self‐control in the scanner task, we did not find hypothesized, statistically significant group differences in dlPFC activity during successful self‐control trials in a whole‐brain analysis. Instead, we observed relative deactivation across multiple brain regions in NSC relative to SC, including, but not limited to, areas that are involved in processing of subjective values such as vmPFC. One possible alternative hypothesis supported by our data thus is that SC do not rely on more intensive executive processing indicated by higher dlPFC activity to downregulate subjective value in self‐control situations, they simply perceive less intensive subjective value for “tempting” food items to begin with. Another alternative explanation is that this null finding is due to power limitations in our data, given that only 15 participants (compared to 19 in the original sample) qualified as SC. In other words, there is a possibility that positive activations in dlPFC during self‐control are simply more subtle than the resulting deactivation in value‐related areas. Although we cannot conclusively disentangle these contradictory ideas, note that we exclusively found negative (although nonsignificant) coefficients within dlPFC in this sample.

Next, we followed procedures reported by Hare et al. (2009) to examine the role of dlPFC in self‐control in terms of its functional connectivity with brain activity in vmPFC. Since we were unable to identify a functionally defined dlPFC cluster in which average activity was involved in self‐control in the replication sample, we relied on a meta‐analytically defined map from www.neurosynth.org (Yarkoni et al., 2011) associated with the term “self‐control” and intersected it with an anatomical, left dlPFC mask. Our analyses which fully replicated the original work by focusing exclusively on processes in participants who were relatively more successful SC during the scanner task did not replicate the original findings which suggested a negative indirect relationship between dlPFC and vmPFC activity through IFG/BA46 during self‐control. We followed up on this null‐result by rerunning the PPI on the full sample of participants who exercised any self‐control in the scanner task (*N* = 59) to address concerns about statistical power. This path was chosen given the absence of strong theoretical arguments that the mechanisms that drive successful self‐control differ qualitatively (rather than just in intensity) between people who are successful more often and those who are successful relatively less often. Indeed, in this larger sample, we do find some evidence of replication. Stronger still, we found evidence of direct, negative correlations between activity within our meta‐analytic left dlPFC seed and an area within vmPFC, which was hypothesized, but not found by Hare et al. (2009). It is important to note, however, that we simultaneously found evidence for unexpected positive associations between activity in the left dlPFC ROI and another, more dorsal MPFC cluster. Of note here is that the whole‐brain table for this analysis in the original publication revealed a similar positive association with an MPFC cluster in almost the exact same location (see Figure 11). While there was (minimal) overlap between the unexpected MPFC cluster that showed positive functional connectivity with left dlPFC and the vmPFC ROI that was associated with goal value in our sample, we did not find such overlap between the vmPFC cluster that showed the hypothesized negative association with dlPFC. In other words, the first PPI, at best, provides mixed evidence regarding the nature of the relationship between dlPFC and vmPFC activity during self‐control. Hare et al. (2009) proceeded to follow‐up on the lack of a negative direct association between dlPFC and vmPFC in their first PPI by identifying a cluster in BA46 that was negatively associated with dlPFC as the seed region for a second PPI. Following this analysis approach, we were able to replicate the original findings, identifying a cluster in vmPFC that was positively associated the BA46 seed identified in PP1 based on the full replication sample (N=59) and thus indirectly negatively associated with the meta‐analytic dlPFC ROI. In sum, our replication data provides mixed evidence with regards to Hypothesis 4 regarding a negative relationship between dlPFC and dlPFC activity during self‐control.

These mixed results highlight the need for additional work to fully understand the role of the dlPFC in food‐related decision‐making and in theories of self‐control more generally. Overall, our findings are most in line with a conceptualization of self‐control as a simple form of value‐based decision‐making in which different choice attributes (here health and taste considerations) are encoded and integrated in vmPFC according to subjective values of the decision‐maker (Berkman et al., 2017). This contrasts with the model that the findings of Hare gave rise to, wherein longer‐term goals (here health considerations) required dlPFC involvement in order to be effectively integrated into subjective value judgments (Hare et al., 2009).

A frequently voiced explanation for failed replications is that the (cultural) context differed between the original and replication study (Zwaan et al., 2018). In our case, the original study was performed in the United States before 2009 and the replication in the Netherlands, approximately 10 years later. Thus far, we are not aware of any strong theoretical or empirical claims that the brains or fundamental psychological processes surrounding self‐control of US subjects are different from those of Dutch study participants or that the basic neural processes of valuation and self‐control have changed over the past decade. However, what could differ between US and Dutch individuals and what could have changed over the past decade is the role of food and dieting in society, and more specifically, to what extent food choices can generate a self‐control conflict and how people cope with that. This may—in theory—influence the way in which people respond to the task and stimuli. Naturally, for a self‐control dilemma to occur one should have the goal to diet or eat healthy. It could be argued that stronger goal commitment may strengthen attempts of overruling impulses and therefore amplify control‐related responses. Observational studies showed that the prevalence of dieting is higher in Europe than in the United States (Santos et al., 2017) and a large proportion of the Dutch population self‐reports to diet or actively restrain their food intake (de Ridder et al., 2014). This would speak against this being an explanation for the null finding. It should however be noted that self‐reports of dieting and dietary restraint have been shown to be unrelated or weakly related to actual intake (de Ridder et al., 2014; Stice et al., 2004) which casts doubt on this measure being a reliable proxy of goal strength. We cannot rule out but we also cannot support that goal commitment was stronger for the successful SC in the original study compared to the current replication study.

Another important conclusion from this project is that analytical flexibility can influence fMRI results. Specifically, for H1 and H2 we presented two sets of results produced using two different analysis strategies. While the overall patterns of results remained similar, increasing confidence in the directionality of effects, effect sizes differed significantly. This has important implications for follow‐up research which may rely on existing work for power calculations. Previous work has shown that not only analytical flexibility but also different preprocessing approaches to the fMRI data (e.g., different software packages and varying parameters) may affect task‐based fMRI results (Bowring et al., 2022; Mikl et al., 2008; Triana et al., 2020). In this replication study we employed a state‐of‐the‐art, standardized, and optimized preprocessing pipeline provided by fMRIprep, which was not available to the authors of the original study (Esteban, Markiewicz, et al., 2018). As much as possible, we chose parameters similar to those used in the original study (e.g., the same smoothing kernel). Though submitting the data through different preprocessing pipelines was outside of the scope of the current study, we acknowledge that doing so could potentially further inform the field about the (in)variability of individual results to specific choices made by the researchers. Unpreprocessed data for this project is available on OpenNeuro and would support such an investigation for those interested.

### 4.1 Impact on theory

Our findings are relevant for future theorizing on self‐control. Specifically, this replication data set supports the conceptualization of self‐control as either a very simple form of value‐based decision‐making (Berkman et al., 2017) or as automatic “effortless” self‐control (Gillebaart & de Ridder, 2015) rather than a dual‐system which involves conscious effortful control.

In psychology, self‐control has traditionally been explained with dual‐system theories (e.g., Hofmann et al., 2008; Metcalfe & Mischel, 1999). These theories are characterized by the notion of two (competing) systems for processing information, namely a “hot”/automatic/impulsive system and a “cold”/rational/reflective system. According to these dual‐system models, self‐control is successful when the impulses arising from the “hot” system are overcome and, consequently, behavior is in line with long‐term goals. In this traditional approach, the dilemma first must be identified and, subsequently, effortful and conscious inhibition is required to overcome it (Fujita, 2011). A neurobiological parallel to these dual‐system models has been proposed in which self‐control involves a balance between brain regions representing the reward, salience and emotional value of a stimulus and prefrontal regions associated with (effortful) inhibition and cognitive control (Heatherton & Wagner, 2011). In this traditional perspective, effortful and conscious impulse inhibition is a necessary or defining feature of (successful) self‐control.

A major criticism of this traditional perspective is that successful self‐control does not always require effortful inhibition or conscious control. It has been proposed that there are many different routes to self‐control, only some of which involve effortful inhibition (Fujita, 2011). Research has indicated that people can automate goal‐striving behaviors in response to contextual cues (Bargh et al., 2001; Chartrand & Bargh, 1996). For instance, providing cues related to the long‐term goal (e.g., dieting cues) promotes goal‐congruent choices through goal priming (Fishbach et al., 2003; Papies, 2016; Van der Laan et al., 2017), which is thought to occur without requiring conscious deliberation or effort. Further, by systematically repeating (healthy) behaviors (healthy) habits can be created. It has been shown that successful SC do not necessarily exert more effort; they perform healthy behaviors automatical because of healthy habits (Galla & Duckworth, 2015; Gillebaart, 2018).

This has led to alternative conceptualizations of self‐control which do not include or at least attenuate the role of effortful inhibition. As mentioned, recently, successful self‐control has been conceptualized as being at least partly an automatic process in which responses to environmental cues that are routinized (or automatically triggered) in the direction that is in line with their long‐term goals (Fujita, 2011; Gillebaart, 2018). A second theory, which recently has gained more traction, is to consider self‐control as a simple value‐based choice (Berkman et al., 2017). Value‐based decision‐making involves choosing an option from a set based on its relative subjective value. This process involves calculating a value for each option by evaluating various attributes—gains (e.g., improved health) and costs (e.g., less food enjoyment), assigning weights to these attributes, and enacting the most valued option. It should be noted that this is a dynamic process. That is, the weight of each attribute is sensitive to attentional shifts (e.g., being explicitly guided toward certain attributes like health), contextual effects and framing of the choice set. Within this conceptualization of self‐control, there is nothing special about long‐term goals: attributes related to short‐ and long‐term goals treated similar in this equation though the relative weights may be different based on the aforementioned factors. This discussion in psychology intersects with the ongoing debate in decision neuroscience and temporal discounting where Kable and Glimcher (2007) suggested there is one common valuation in vmPFC while McClure et al. (2004) suggested that separate neural systems encode value for immediate versus longer‐term attributes.

The study of Hare conceptualizes self‐control as a value‐based decision (H1, H2) but in line with traditional dual‐system models it still posi that there are dual motives and that the future part is “special”: integrating longer‐term considerations into the value system, that is, changing the weight of long‐term attributes, requires involvement from control‐related areas (i.e., the dlPFC; H3, H4). Their hypothesis about the role of the dlPFC had its basis in the role of dlPFC in cognitive control and emotion regulation. The authors speculated that vmPFC originally evolved to predict the short‐term value of stimuli and that humans developed the ability to incorporate long‐term considerations into values by giving structures such as the dlPFC the ability to modulate this value.

Our mixed findings regarding dlPFC involvement highlight the need for more research to understand the role of dlPFC in assigning weight to these longer‐term consequences. The replication results rather point to the conceptualization of self‐control as either automatic and “effortless” or as a (simple) form of value‐based decision‐making. At a minimum, our results support the idea that that it is not the dlPFC that is responsible for increasing the weight of the longer‐term attributes into the choice. In support of the latter: when comparing successful to unsuccessful trials that required self‐control in all participants, we observed a deactivation of vmPFC, which suggests that successful self‐control in this sample may be driven by a weaker subjective value for a given food item rather than by more intensive control driven by dlPFC. The finding, that in successful SC, vmPFC reflects health ratings, even though dlPFC is not active, suggests that dlPFC activation is not needed to incorporate health into the vmPFC value signal. Thus indeed, in line with the proposition of self‐control as a simple form of value‐based decision‐making (Berkman et al., 2017), decisions may just be the result of multiple single value‐calculations.

## AUTHOR CONTRIBUTIONS

Christin Scholz: Conceptualization, formal analysis, visualization, data curation, validation, writing – original draft, writing – review and editing. Han Y. Chan: Formal analysis, writing – original draft. Russell A. Poldrack: Conceptualization, writing – review and editing. Denise T. D. de Ridder: Conceptualization, methodology, writing – review and editing. Ale Smidts: Conceptualization, methodology, writing – review and editing. Laura Nynke van der Laan: Conceptualization, methodology, validation, investigation, writing – original draft, writing – review and editing, project administration, funding acquisition.

## FUNDING INFORMATION

This work is supported by a governmental research grant: the call for Replication studies of the Dutch Science Foundation (NWO) (grant number: 401.16.023). The funding source had no role in the design of this study, the data collection, analysis and interpretation of data; in the writing of the report; and in the decision to submit the article for publication.

## CONFLICT OF INTEREST

The authors declare no conflicts of interest.

## Supporting information


**Appendix S1** Supporting Information

## Data Availability

The data are publicly available at OpenNeuro (accession number: ds002643).

## Footnotes

^1^
2012 citations, retrieved on February 9, 2022.

^2^
In the supplemental materials of the original study, it was mentioned that also “individuals who self‐reported no current monitoring of their diet” were included.

^3^
Recoded to −2 = very unhealthy/very bad and 2 = very healthy/very good.
