# Supplementary material for: Can we have a second helping? A preregistered direct replication study on the neurobiological mechanisms underlying self‐control
Source: Hum Brain Mapp. 2022 Sep 9;43(16):4995–5016. doi: 10.1002/hbm.26065 (PMC9582371; doi:10.1002/hbm.26065)
Supplement: Supplementary file 1 — Appendix S1 Supporting Information [file HBM-43-4995-s001.docx]

Supplementary Materials

Can we have a second helping? A preregistered direct replication study on the neurobiological mechanisms underlying self-control

C. Scholz^1^, H.Y. Chan^1^, R.A. Poldrack^2^, D.T.D. de Ridder^3^, A. Smidts^4^ & L. N. van der Laan^5*^

^1^ Amsterdam School of Communication Research, University of Amsterdam. Address: Nieuwe achtergracht 166, 1018 WV Amsterdam, The Netherlands. Email: [c.scholz@uva.nl](mailto:c.scholz@uva.nl), h.y.chan@uva.nl

^2^ Department of Psychology, Stanford University. Address: 450 Jane Stanford Way, Building 420, Stanford, CA 94305, United States of America. Email: poldrack@stanford.edu

^3^ Department of Social, Health and Organisational Psychology, Utrecht University. Address: Heidelberglaan 1, 3584CS Utrecht, The Netherlands. Email: d.t.d.deridder@uu.nl

^4^ Rotterdam School of Management, Erasmus University Rotterdam. Address: Burgemeester oudlaan 50, 3062 PA Rotterdam, The Netherlands. Email: asmidts@rsm.nl

^5^ Department of Communication and Cognition, Tilburg University. Address: P.O. Box 90153, 5000 LE Tilburg, The Netherlands. Email: [l.n.vdlaan@tilburguniversity.edu](mailto:l.n.vdlaan@tilburguniversity.edu).

Author Note

L. N. van der Laan [
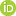
](https://orcid.org/0000-0001-6905-1832) https://orcid.org/0000-0003-4307-0888

Correspondence concerning this article should be addressed to L. N. van der Laan, Department of Communication and Cognition, Tilburg University, P.O. Box 90153, 5000 LE Tilburg, The Netherlands. Email: [l.n.vdlaan@tilburguniversity.edu](mailto:l.n.vdlaan@tilburguniversity.edu).

**Tables**

Table S1. Characteristics of the stimulus set in the original study as well as this replication study (including results of online and lab pilot studies and the fMRI study). A more elaborate description of procedures and results of the pilot studies can be found on OSF (https://osf.io/qzyxm/).

|  | **Original study** | | | | **Replication study** | | | | | | | | | | | | |
| --- | --- | --- | --- | --- | --- | --- | --- | --- | --- | --- | --- | --- | --- | --- | --- | --- | --- |
|  |  |  |  |  | **(Online) pilot** | | | **(Lab) pilot** | | | | **fMRI study** | | | | |  |
| Health rating Mean (SD) | -0.29 (1.29) | | | | -0.34 (1.25) | | | -0.35 (1.31) | | | | -0.36 (1.24) | | | | |  |
| Taste rating Mean (SD) | 0.57 (0.52) | | | | 0.67 (0.51) | | | 0.57 (0.66) | | | | 0.57 (0.59) | | | | |  |
| Self-control required trials (unliked healthy/liked unhealthy) | 34.8% (success: 45.3%) | | | | 39.8%^1^ | | | 39.2% (success: 27.6%) | | | | 37.1% (success: 23.8%) | | | | |  |
|  |  | | | |  | | |  | | | |  | | | |  |  |
| Stimulus/trial category | % trials | % yes | Health  rating | Taste  rating | % trials | Health  rating | Taste  rating | % trials | % yes | Health  rating | Taste  rating | % trials | % yes | Health  rating | Taste  rating |  |  |
| Liked healthy (health & taste > 0) | 21.5% | 74.0% | 1.59 (0.27) | 1.36 (0.31) | 21.9% | 1.44 (0.37) | 1.31 (0.29) | 21.4% | 83.8% | 1.51 (0.31) | 1.37 (0.23) | 21.0% | 80.5% | 1.47  (0.50) | 1.34  (0.47) |  |  |
| Liked unhealthy (health < 0, taste > 0) | 29.8% | 41.7% | -1.58 (0.28) | 1.37 (0.23) | 35.8% | -1.47 (0.30) | 1.30 (0.23) | 34.6% | 60.8% | -1.54 (0.27) | 1.32 (0.21) | 32.0% | 62.8% | -1.52  (0.50) | 1.33  (0.47) |  |  |
| Unliked healthy (health > 0, taste < 0) | 5.0% | 14.6% | 1.42 (0.36) | -1.29 (0.32) | 4.0% | 1.29 (0.42) | -1.34 (0.44) | 4.6% | 0.0% | 1.54 (0.39) | -1.33 (0.41) | 4.2% | 4.8% | 1.39  (0.49) | -1.36  (0.48) |  |  |
| Unliked unhealthy (health < 0, taste < 0) | 10.1% | 8.7% | -1.55 (0.37) | -1.33 (0.31) | 8.4% | -1.57 (0.41) | -1.19 (0.28) | 11.2% | 2.3% | -1.47 (0.33) | -1.28 (0.29) | 3.0% | 0.8% | -1.54  (0.50) | -2.00  (0.00) |  |  |

^1^The aim of the online pilot was to get a first estimate of the perceived healthiness and tastiness of the stimulus set and the number of stimuli rated by each participant was 20 (instead of 50 as in the original study). Because the number of choices was very different from the original study, we do not report the choice behavior of the online pilot.

Table S2. Stimulus sets of the original and replication studies. The F4H stimuli can be downloaded from https://osf.io/cx7tp/.

| Hare 2009 image code |  | Original study image | Replication study image | F4H image code |
| --- | --- | --- | --- | --- |
| 1 | Banana | 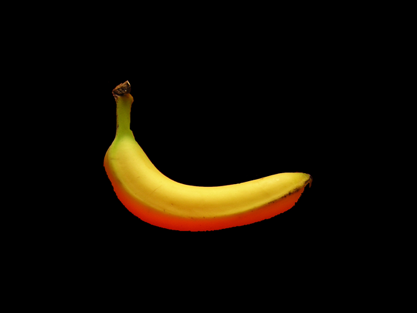 | 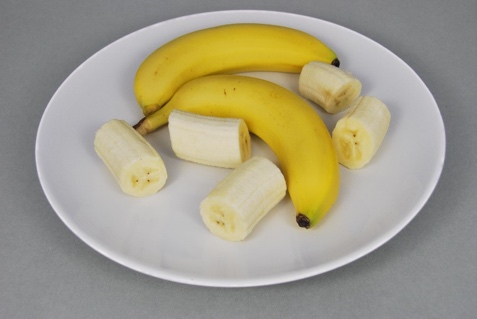 | 81 |
| 2 | Blueberry yogurt | 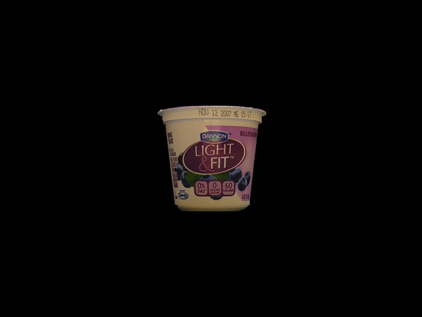 | 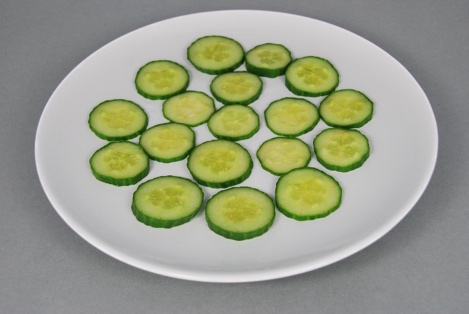 | 65 |
| 3 | Broccoli and cauliflower | 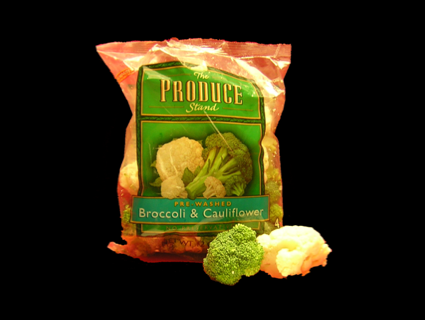 | 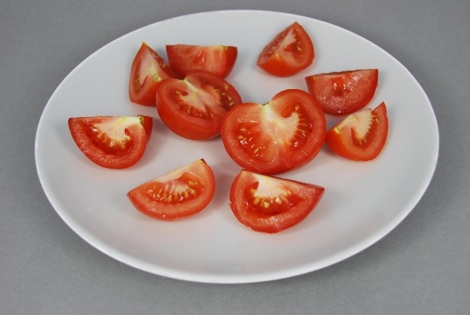 | 171 |
| 4 | Carrots | 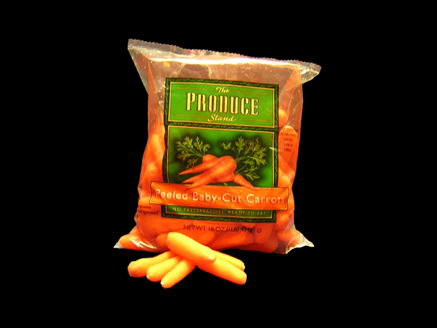 | 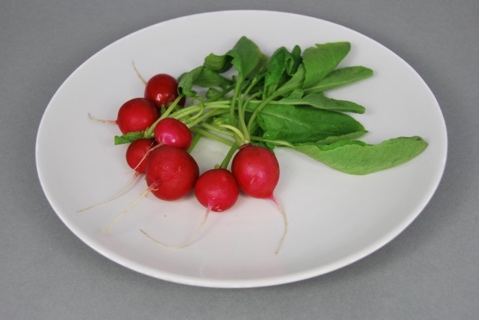 | 53 |
| 5 | Celery | 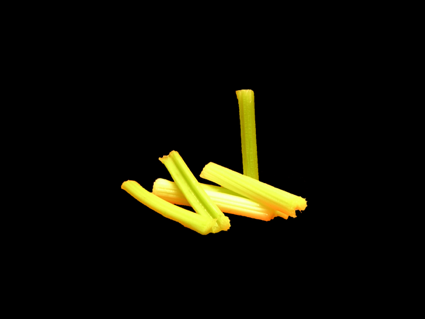 | 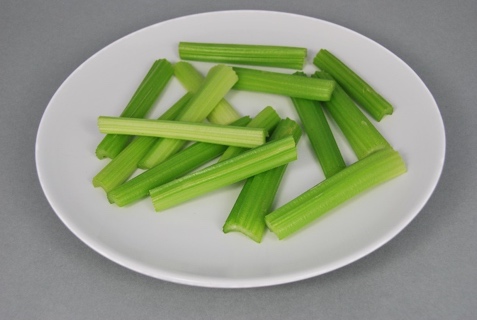 | 74 |
| 6 | Cranberries | 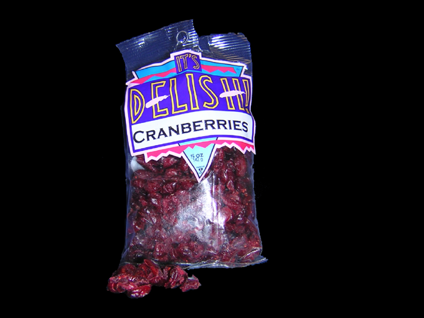 | 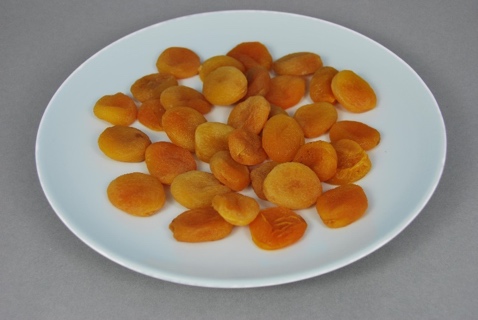 | 213 |
| 7 | Fat free raspberry sorbet | 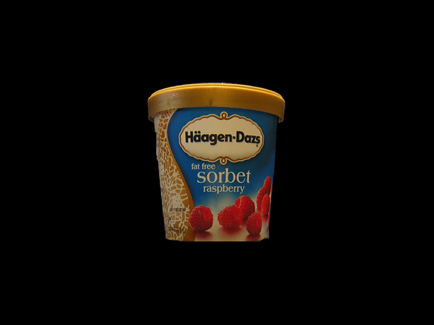 | 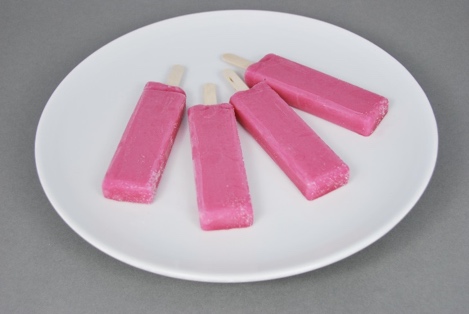 | 154 |
| 8 | Vanilla frozen yogurt | 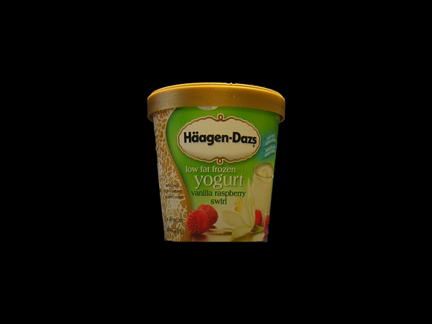 | 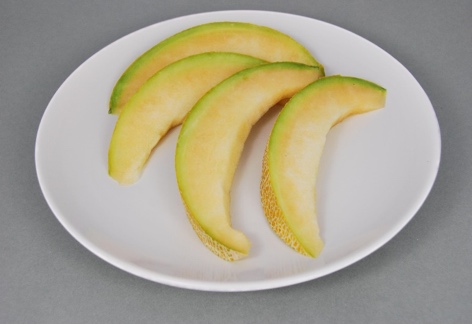 | 91 |
| 9 | Granny smith apple | 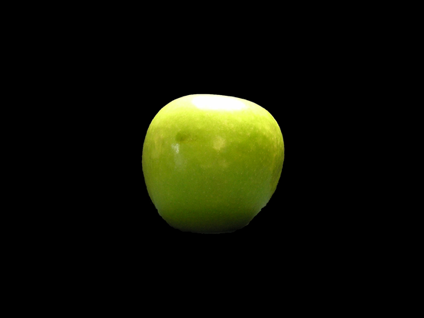 | 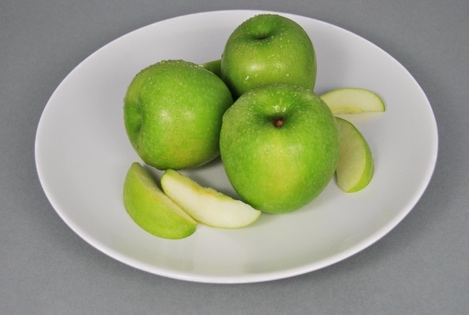 | 142 |
| 10 | Mixed berry yogurt | 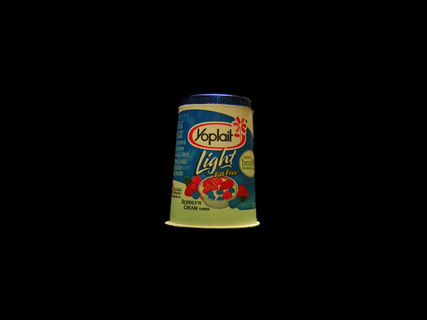 | 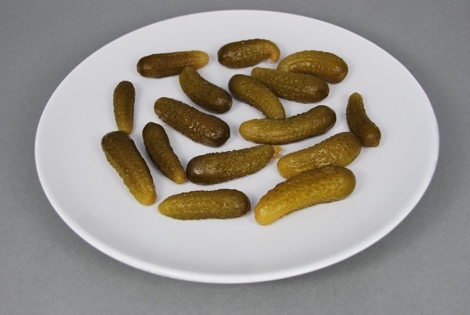 | 69 |
| 11 | Orange | 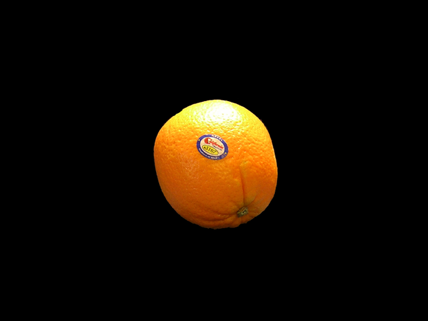 | 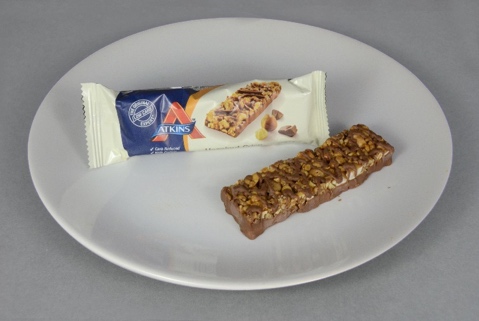 | NA (new image) |
| 12 | Orange flavored jello | 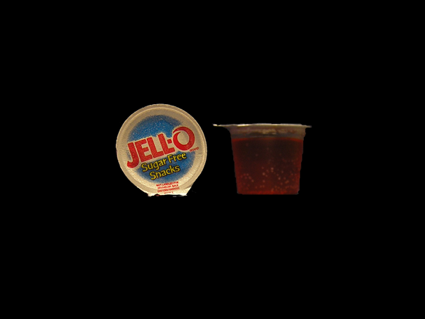 | 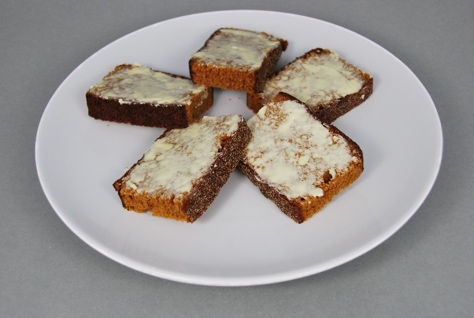 | 34 |
| 13 | Raisins | 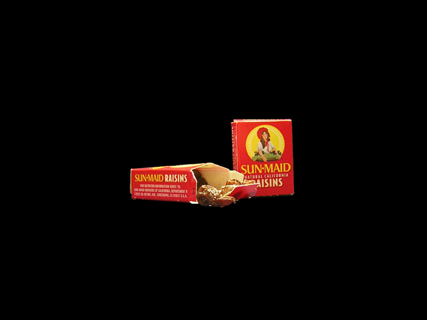 | 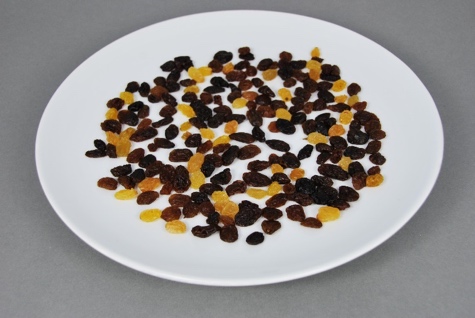 | 89 |
| 14 | Red delicious apple | 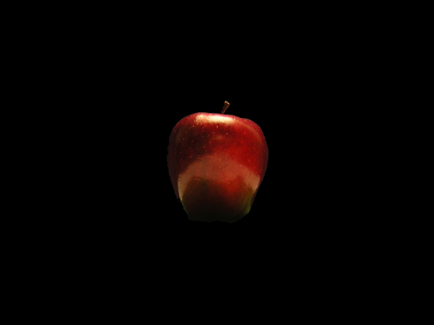 | 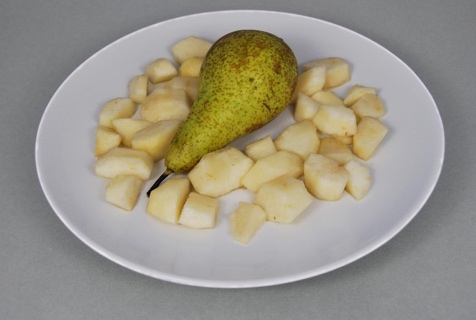 | 153 |
| 15 | Red grapes | 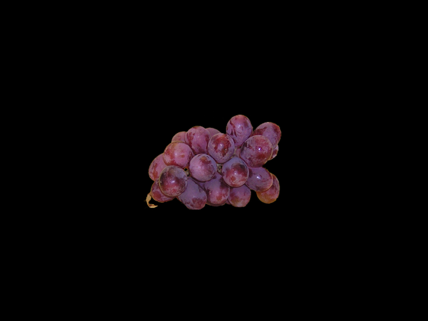 | 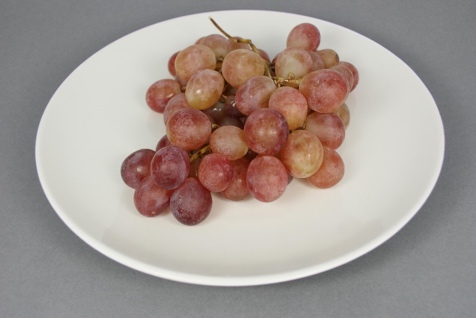 | 259 |
| 16 | South beach diet wheat crackers | 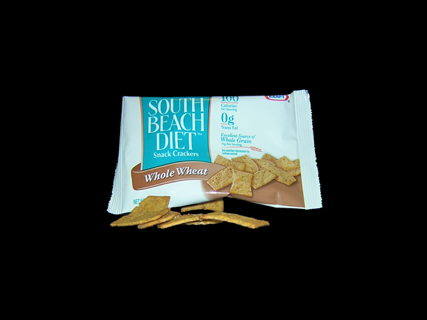 | 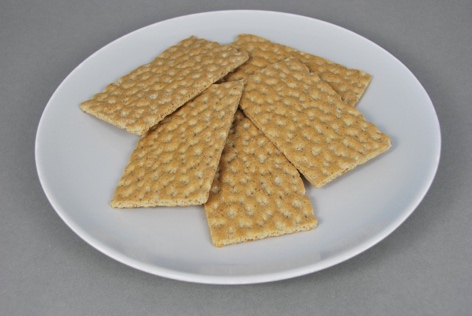 | 72 |
| 17 | South beach diet bar granola | 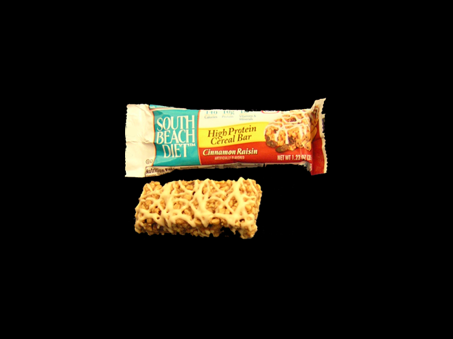 | 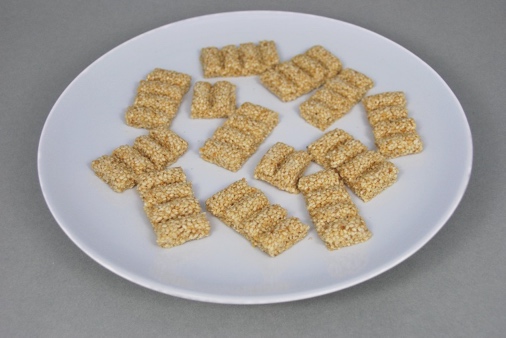 | 337 |
| 18 | Slim fast chocolate shake | 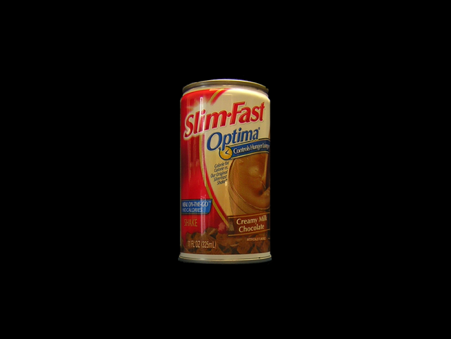 | 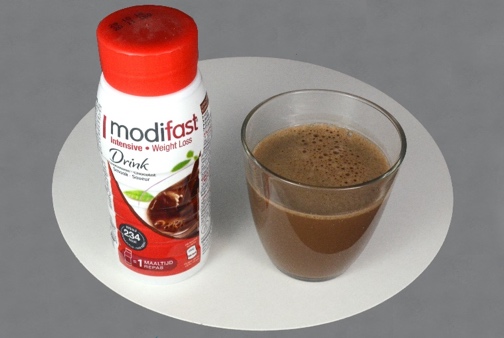 | NA (new image) |
| 19 | Slim fast vanilla shake | 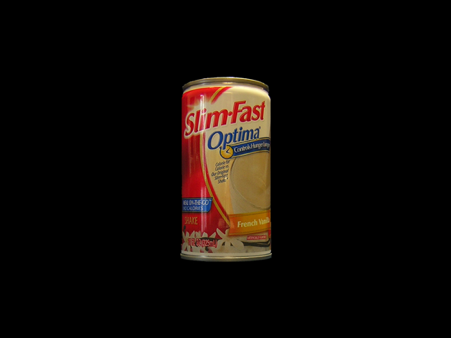 | 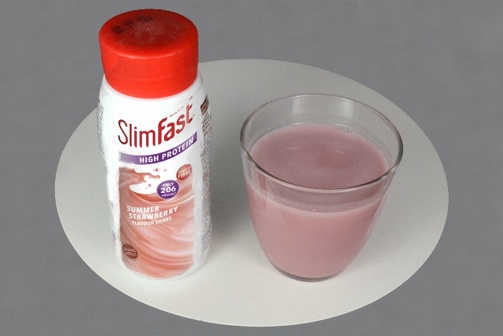 | NA (new image) |
| 20 | Special K snack bar | 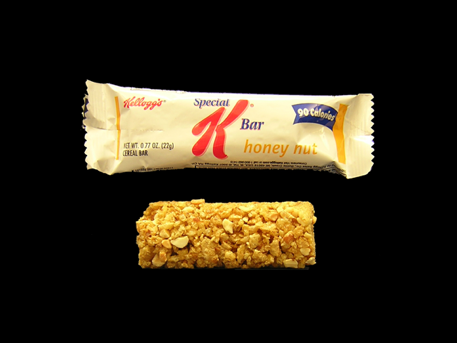 | 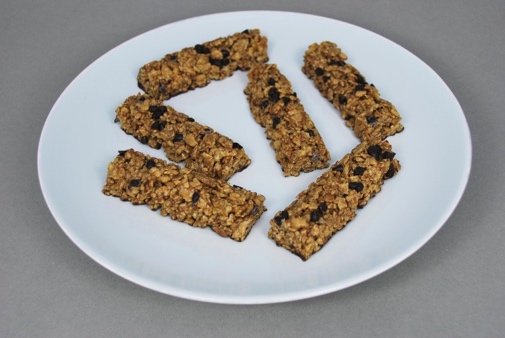 | 336 |
| 21 | Strawberries | 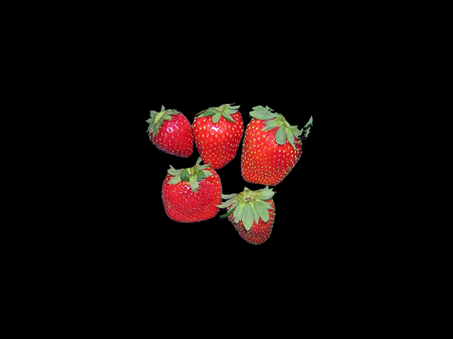 | 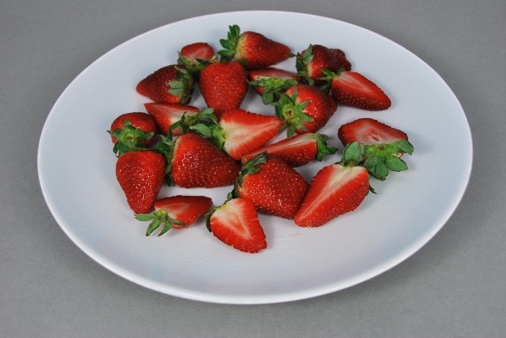 | 144 |
| 22 | Wheat crisp crackers | 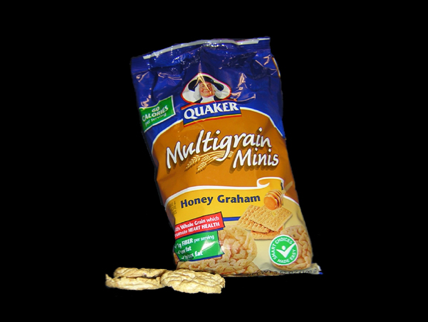 | 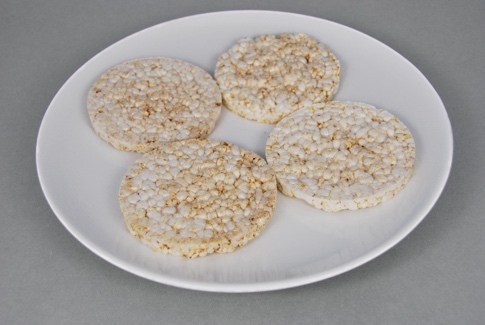 | 64 |
| 23 | White grapes | 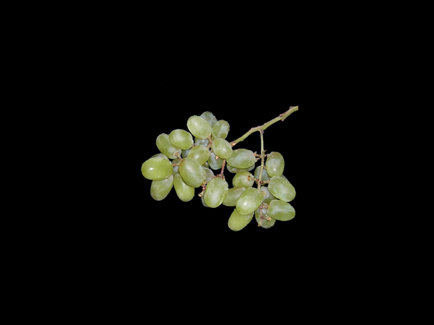 | 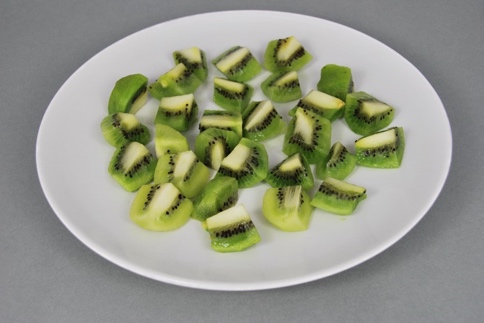 | 90 |
| 24 | Weight watchers chocolate brownie | 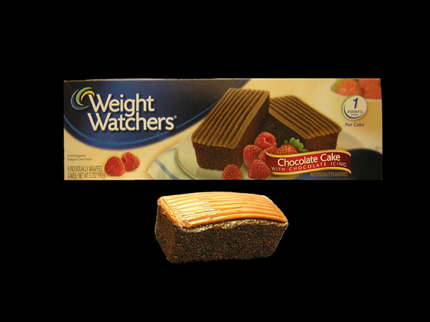 | 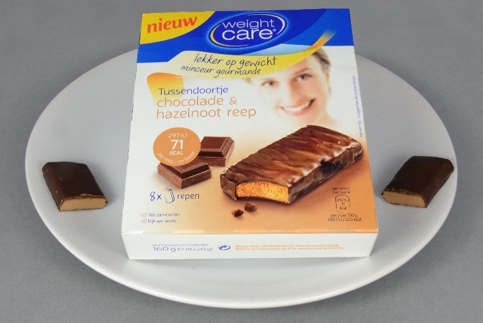 | NA (new image) |
| 25 | Weight watchers blueberry muffin | 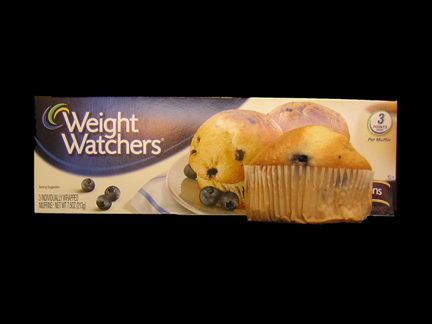 | 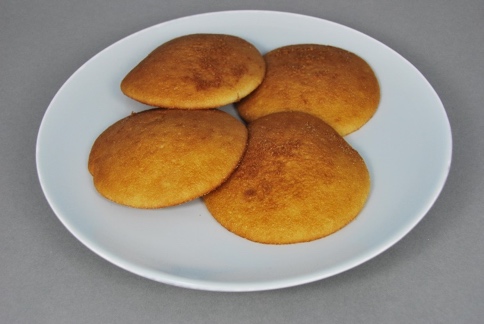 | 82 |
| 26 | 100 Grand candy bar | 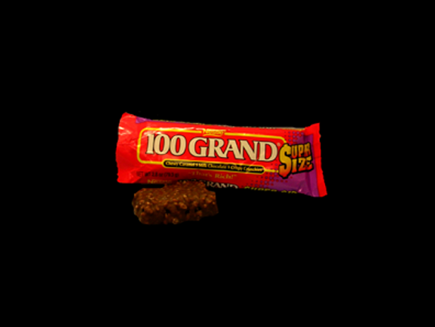 | 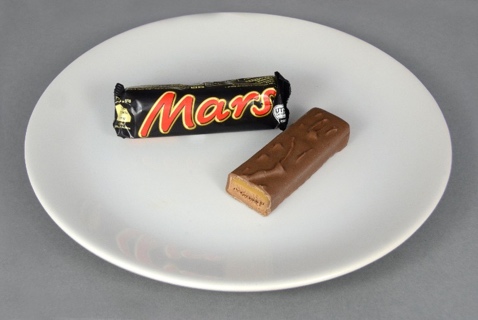 | NA (new image) |
| 27 | Chips Ahoy cookies | 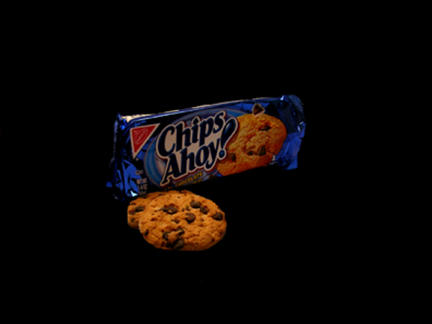 | 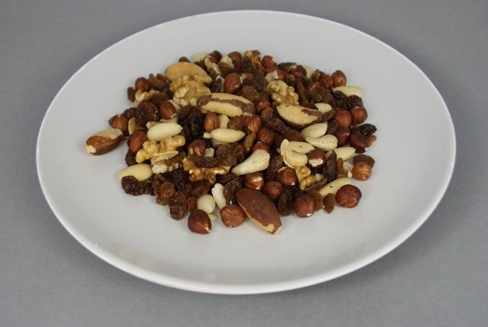 | 326 |
| 28 | Doritos ranch chips | 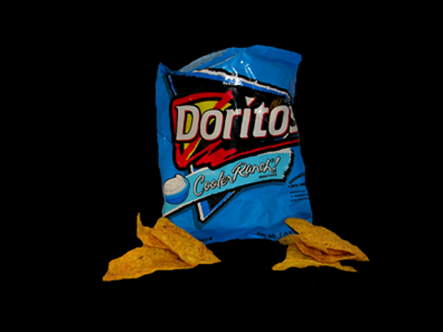 | 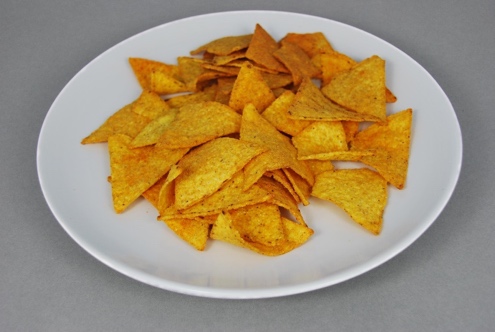 | 24 |
| 29 | Famous Amos cookies (chocolate chip) | 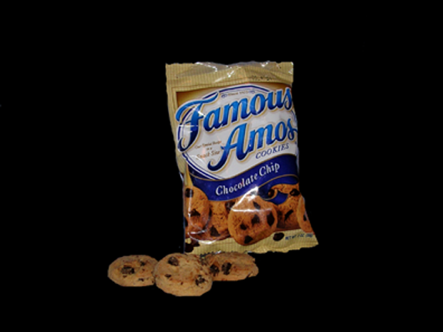 | 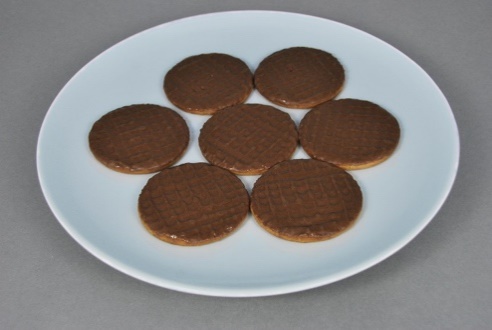 | 35 |
| 30 | Flaming Cheetos cheese puffs | 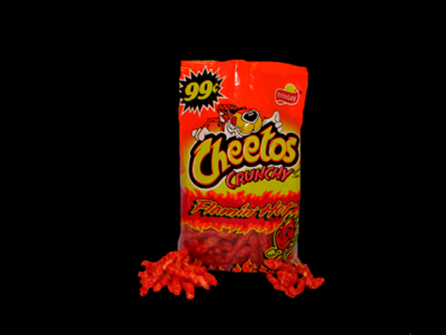 | 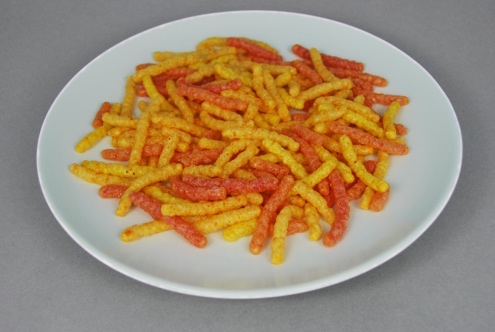 | 139 |
| 31 | Ghiradelli milk chocolate | 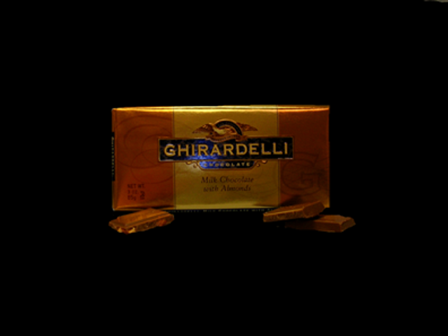 | 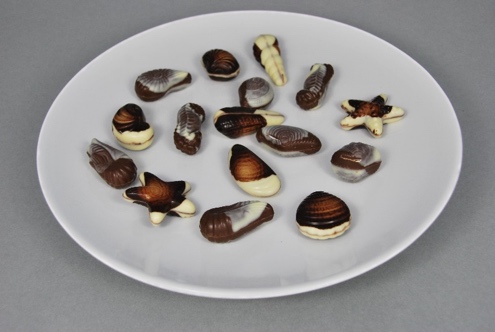 | 116 |
| 32 | Hostess HoHos | 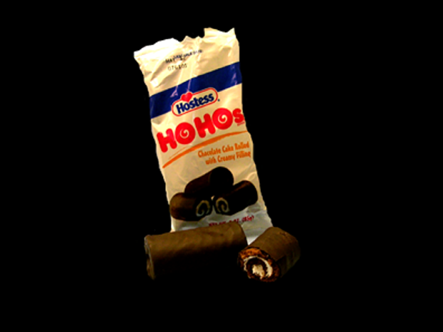 | 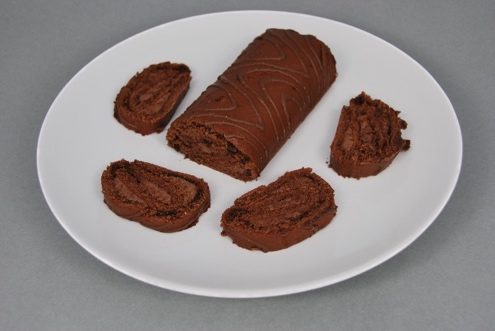 | 353 |
| 33 | KitKat candy bar | 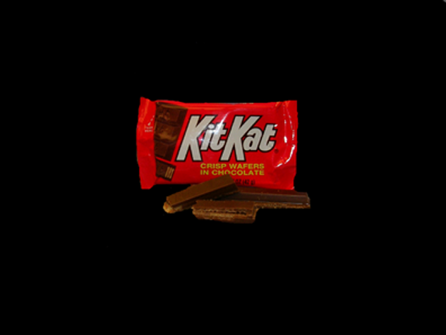 | 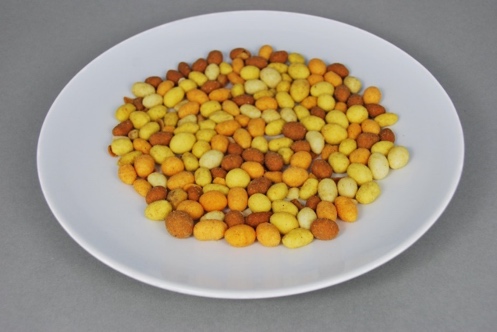 | 2 |
| 34 | Lindt swiss chocolate bar | 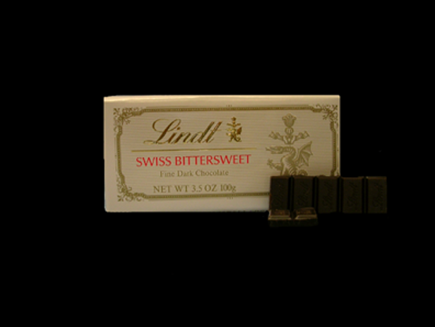 | 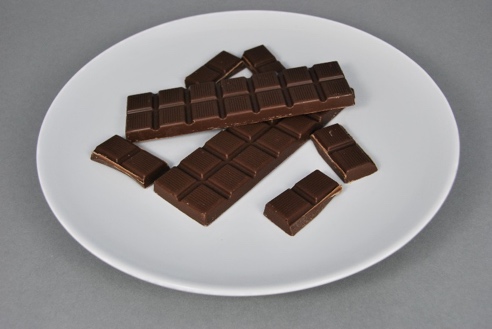 | 32 |
| 35 | MrsFields cholate chip cookie | 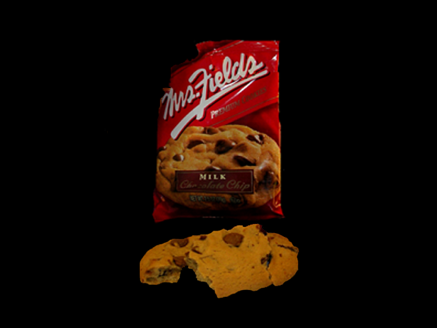 | 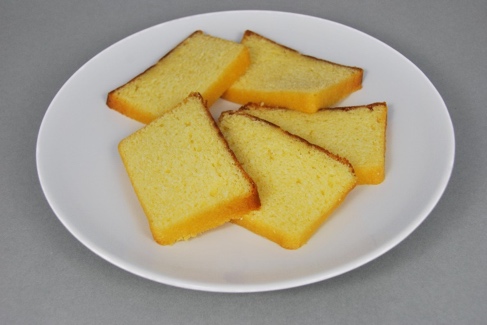 | 118 |
| 36 | Oreo cookies | 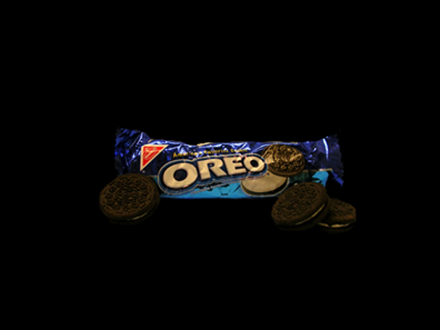 | 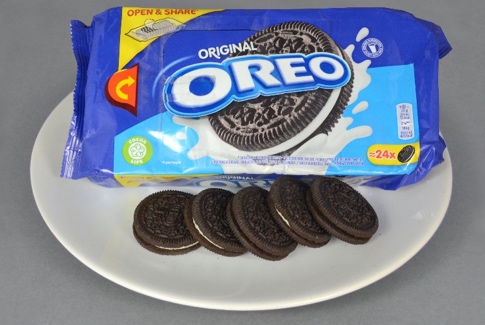 | NA (new image) |
| 37 | Reeses Peanut butter cups | 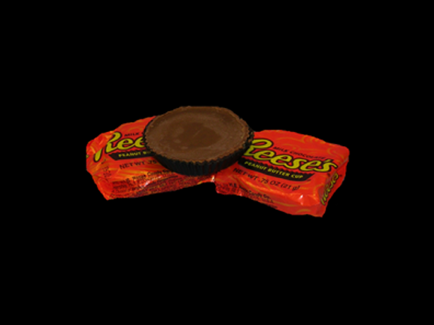 | 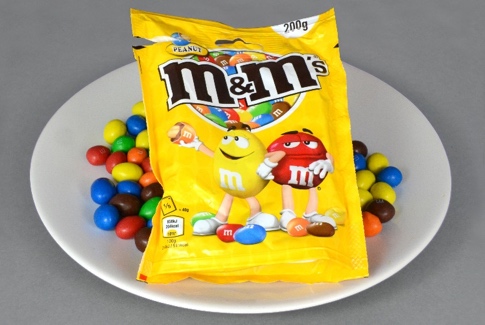 | NA (new image) |
| 38 | RiceKrispyTreat | 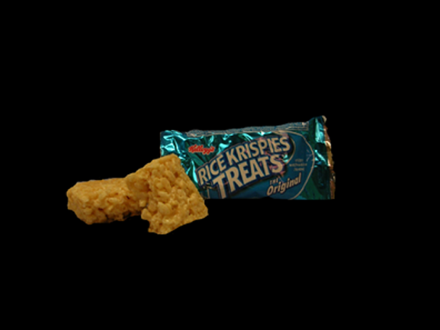 | 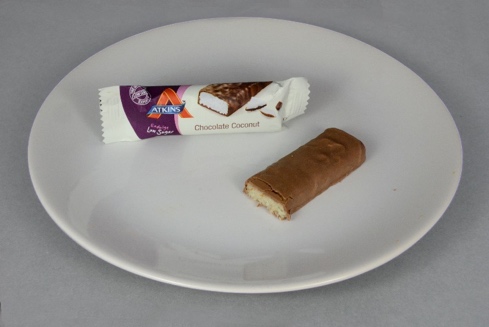 | NA (new image) |
| 39 | Butterfinger candy bar | 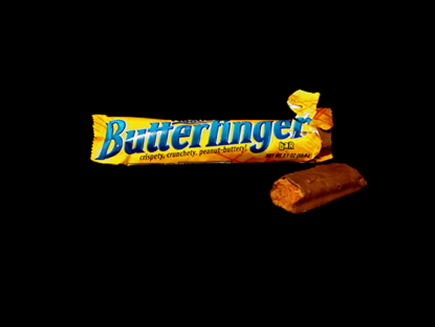 | 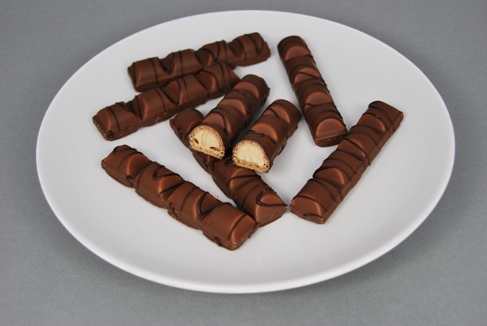 | 104 |
| 40 | Cherry ice cream | 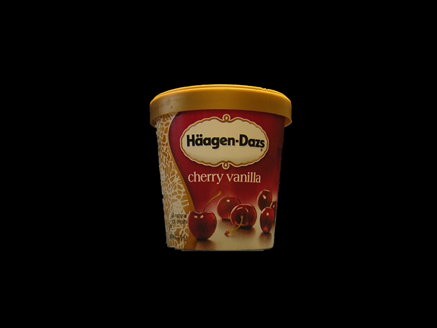 | 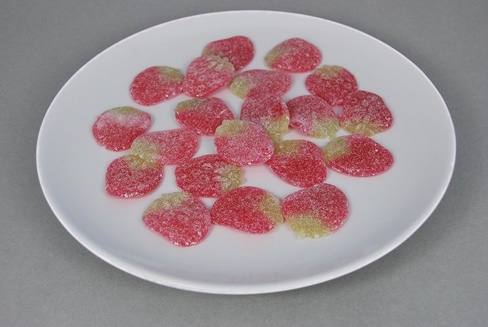 | 40 |
| 41 | Cookies and cream ice cream | 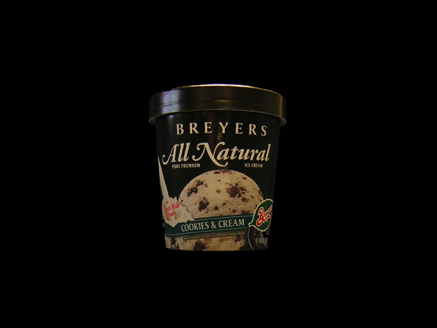 | 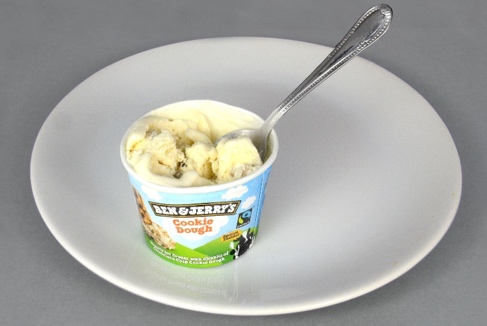 | NA (new image) |
| 42 | Milano cookies | 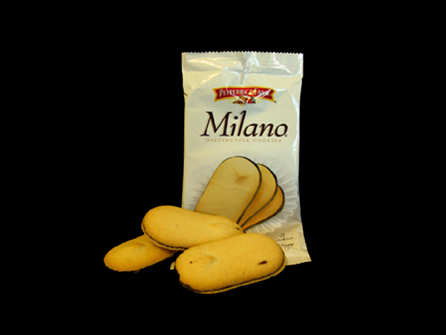 | 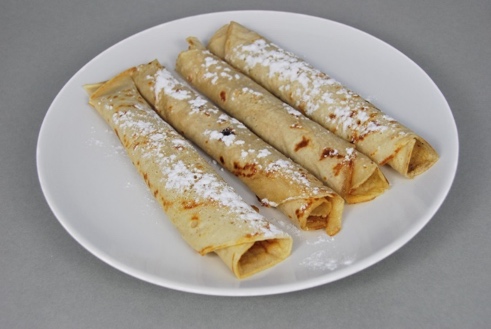 | 101 |
| 43 | Ice cream sandwich | 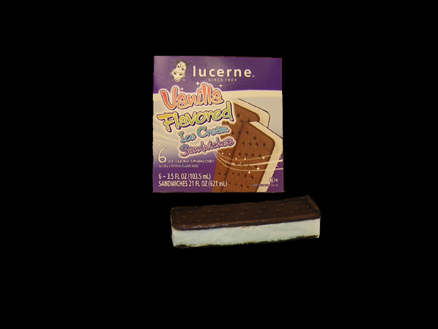 | 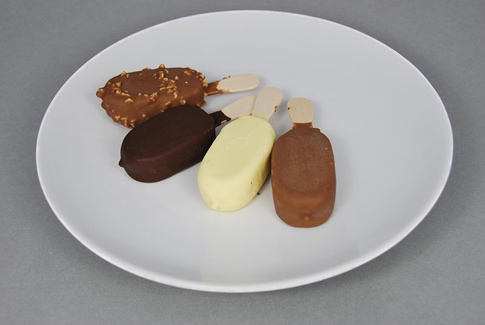 | 119 |
| 44 | Keebler fudge stripes cookies | 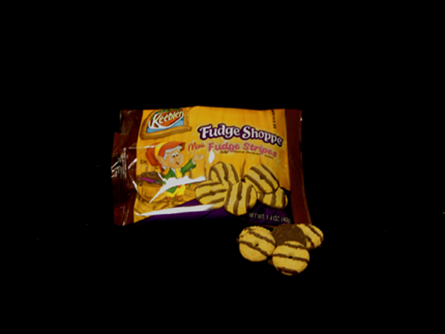 | 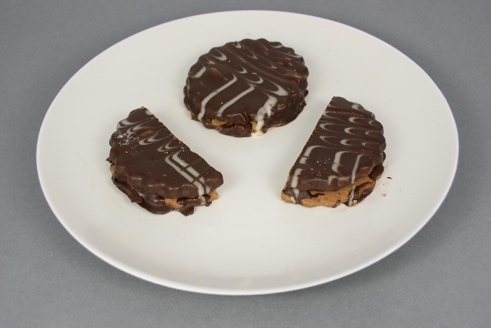 | 255 |
| 45 | Keebler rainbow cookies (chocchip) | 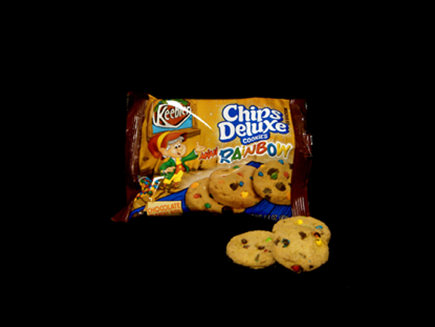 | 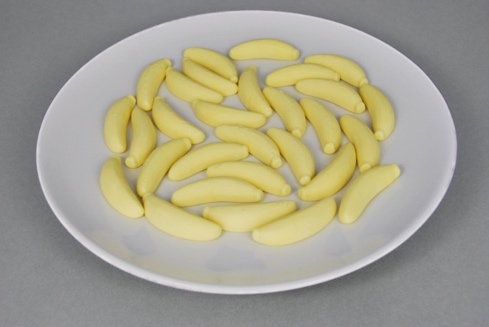 | 45 |
| 46 | Lays classic potato chips | 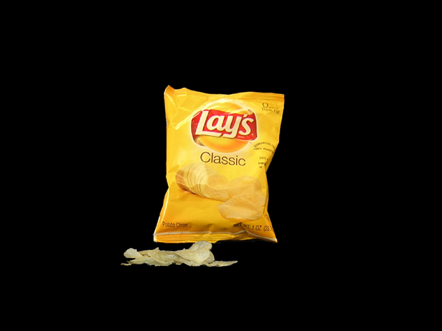 | 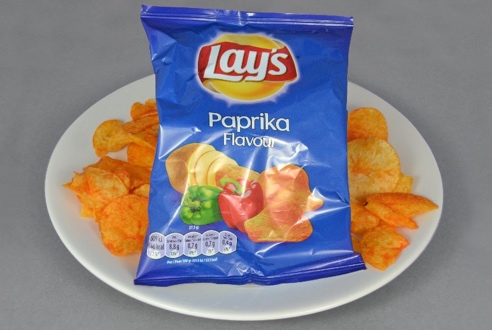 | NA (new image) |
| 47 | Lays ruffles potato chips | 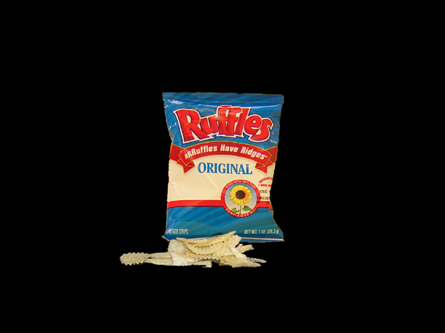 | 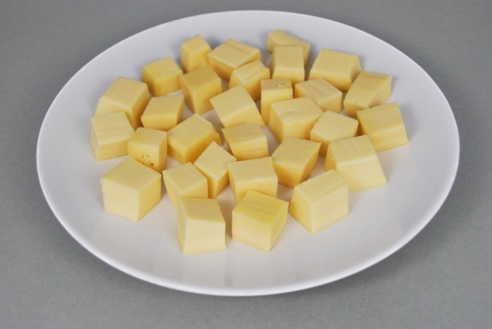 | 8 |
| 48 | Hostess strussel cakes | 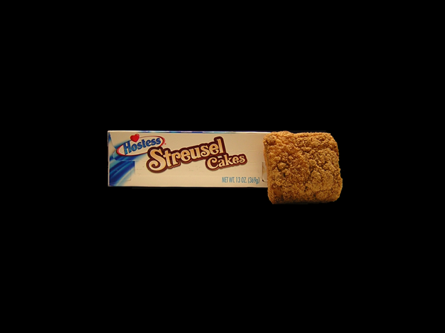 | 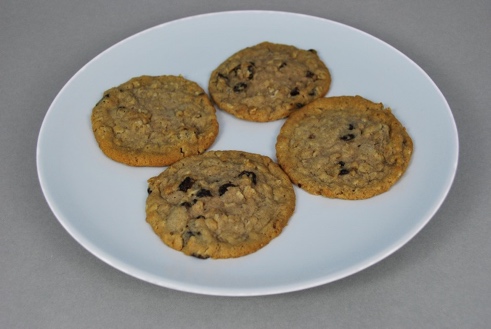 | 235 |
| 49 | Toblerone chocolate bar | 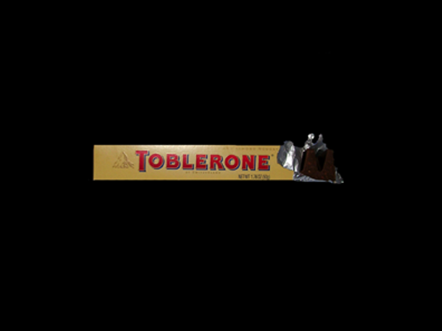 | 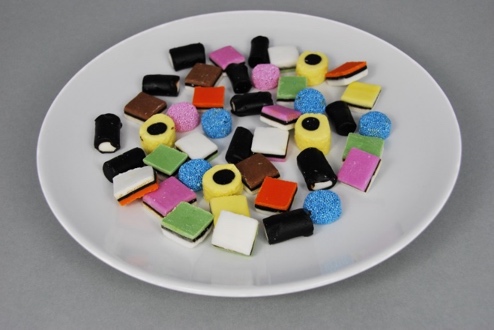 | 48 |
| 50 | Twix caramel and chocolate bar | 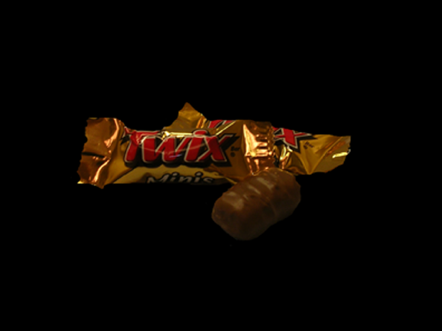 |  | 29 |

Table S3. Average reaction times with standard errors (SEM) by rating and self-control group membership

| **Food rating** | **SC group** | **NSC Group** |
| --- | --- | --- |
| Disliked-Unhealthy | 990 (SEM=110) | 1228 (SEM=67) |
| Disliked-Healthy | 1692 (SEM=94) | 1532 (SEM=52) |
| Liked-Unhealthy | 1942 (SEM=47) | 1537 (SEM=20) |
| Liked-Healthy | 1624 (SEM=60) | 1414 (SEM=23) |

Table S4. Whole Brain Analysis GLM1. Brain regions in which activity correlated more strongly with goal values during decision trials than during taste trials, thresholded at p < .005 uncorrected. * marks clusters with significant voxels thresholded at FDR p < .05.

| Region | Hemis-phere | MNI Coordinates | | | Cluster average |
| --- | --- | --- | --- | --- | --- |
|  |  | X | Y | Z |  |
| Medial prefrontal cortex* | R | 3 | 39 | -8.7 | 3.56 |
| Insula* | R | 30 | 36 | -12 | 3.21 |
| Superior frontal gyrus | L | -18 | 12 | 70.5 | 2.95 |
| Cerebellum | R | 45 | -57 | -45 | 3.07 |
| Frontal pole | L | -36 | 60 | 14.4 | 3.03 |
| Precentral gyrus | R | 36 | -15 | 57.3 | -3.03 |
| Postcentral gyrus | R | 54 | -18 | 50.7 | -3.01 |
| Anterior cingulate* | - | 0 | 15 | 24.3 | 3.35 |
| Superior temporal gyrus | R | 57 | -15 | -2.1 | 2.99 |

Table S5. Means and standard deviations per goal value level for Figures 5B-D.

| cond | mean | sd | ROI |
| --- | --- | --- | --- |
| Strong No | -0.9917277 | 2.50094353 | VMPFC (peak voxels) |
| No | -1.1779471 | 1.6774849 | VMPFC (peak voxels) |
| Neutral | -0.5552562 | 1.64612215 | VMPFC (peak voxels) |
| Yes | 0.80273636 | 1.717442 | VMPFC (peak voxels) |
| Strong Yes | 1.74112325 | 2.67081106 | VMPFC (peak voxels) |
| Yes | 0.00145729 | 0.0059497 | VMPFC (averaged) |
| Neutral | -0.0013529 | 0.00526019 | VMPFC (averaged) |
| No | -0.0023422 | 0.00659549 | VMPFC (averaged) |
| Strong No | -3.16E-04 | 0.01011161 | VMPFC (averaged) |
| Strong Yes | 0.0061144 | 0.00894514 | VMPFC (averaged) |
| Strong No | 0.01264191 | 2.54034108 | Caudate (peak voxels) |
| No | -0.1462815 | 1.55764979 | Caudate (peak voxels) |
| Neutral | 0.0129301 | 1.33210527 | Caudate (peak voxels) |
| Yes | 0.81222513 | 1.65024095 | Caudate (peak voxels) |
| Strong Yes | 1.09381024 | 2.29186392 | Caudate (peak voxels) |

Table S6. Whole Brain Analysis GLM4. Areas showing stronger responses in successful self-control trials in the SC compared to the NSC group, thresholded at p < .005 uncorrected. Note that no voxels are significant when applying multiple comparison correct at FDR p < .05.

| Region | Hemis-phere | MNI Coordinates | | | Cluster average |
| --- | --- | --- | --- | --- | --- |
|  |  | X | Y | Z |  |
| Posterior cingulate cortex | - | 0 | -18 | 30.9 | -3.20 |
| Ventro-medial prefrontal cortex | L | -6 | 45 | -21.9 | -3.05 |
| Superior temporal pole | L | -30 | 9 | -21.9 | -3.14 |
| Dorsolateral pre-frontal cortex | L | -42 | 9 | 54 | -3.18 |
| Orbitofrontal cortex | R | 6 | -9 | -48.3 | -3.36 |
| Superior frontal gyrus | L | -24 | 27 | 54 | -3.05 |
| Postcentral gyurs | L | -48 | -21 | 63.9 | -2.98 |
| Superior frontal gyrus | L | -15 | 21 | 70.5 | -3.16 |
| Temporal pole | R | 30 | 12 | -21.9 | -3.21 |

Table S7. Whole Brain Analysis PPI 1. Areas showing task related functional connectivity with left DLPFC; thresholded at p<.005 uncorrected, k>=15. Clusters that survive FDR p < .05 thresholding are marked in the rightmost column.

| Region | Hemis-phere | MNI Coordinates | | | Cluster average | FDR p < .05 |
| --- | --- | --- | --- | --- | --- | --- |
|  |  | X | Y | Z |  |  |
| Inferior occipital gyrus | L | -48 | -63 | -12 | -8.31103 | * |
| Dorsomedial prefrontal cortex | R | 3 | 63 | 27.6 | 4.88829 | * |
| Supplemental motor area | L | -6 | 15 | 50.7 | -4.47866 | * |
| Cingulate gyrus | R | 18 | 6 | 27.6 | 3.57951 | * |
| Cerebellum | L | -15 | -33 | -45 | 3.9476 | * |
| Precentral gyrus | L | -33 | -3 | 57.3 | -4.04345 | * |
| Precentral gyrus | L | -48 | 6 | 37.5 | -4.54557 | * |
| Precentral gyrus | R | 42 | 3 | 37.5 | -3.82863 |  |
| Posterior cingulate | L | -27 | -54 | 17.7 | 3.49952 |  |
| Cingulate gyrus | R | 15 | -39 | 44.1 | 3.72561 |  |
| Parahippocampus | L | -36 | -51 | 1.2 | 3.45979 | * |
| Cerebellum | R | 24 | -87 | -25.2 | 4.24194 |  |
| Precuneus | - | -12 | -48 | 50.7 | 3.29455 |  |
| Cerebellum | L | -3 | -48 | -51.6 | 3.35336 |  |
| Cuneus | L | -9 | -75 | 40.8 | -3.60323 |  |
| Middle frontal gyrus | R | 45 | 30 | 24.3 | -3.83285 | * |
| Postcentral gyrus | L | -63 | -3 | 11.1 | 3.23015 |  |
| Superior frontal gyrus | L | -30 | 57 | -12 | -3.19963 | * |
| Superior frontal gyrus | R | 15 | 63 | -18.6 | -3.32415 |  |
| Pallidum | R | 15 | 3 | -2.1 | -3.80387 |  |
| Cerebellum | R | 21 | -33 | -48.3 | 3.12381 |  |
| Cerebellum | - | 0 | -87 | -21.9 | 3.45417 |  |
| Middle frontal gyrus | R | 30 | 0 | 57.3 | -3.4491 |  |
| Postcentral gyrus | L | -48 | -15 | 27.6 | 3.30109 |  |
| Cerebellum | R | 27 | -42 | -51.6 | 3.42456 |  |
| Precuneus | L | -6 | -51 | 63.9 | 2.95213 |  |
| Cerebellum | R | 48 | -72 | -28.5 | 3.37125 |  |
| Superior temporal gyrus | L | -60 | -9 | -5.4 | 3.45116 |  |
| Posterior cingulate | R | 30 | -51 | 17.7 | 3.16141 |  |

Table S8. Whole Brain Analysis PPI 2. Areas showing task related functional connectivity with left IFG; thresholded at p<.005, k>=15. Clusters that survive FDR p < .05 thresholding are marked in the rightmost column.

| Region | Hemis-phere | MNI Coordinates | | | Cluster average | FDR p < .05 |
| --- | --- | --- | --- | --- | --- | --- |
|  |  | X | Y | Z |  |  |
| Fusiform gyrus | L | -51 | -57 | -15.3 | -5.18777 | * |
| Inferior frontal gyrus | L | -48 | 45 | 1.2 | -6.88093 | * |
| Inferior parietal lobe | L | -54 | -48 | 50.7 | -6.68658 | * |
| Middle frontal gyrus | R | 48 | 36 | 30.9 | -5.30025 | * |
| Angular gyrus | R | 36 | -69 | 44.1 | -5.92775 | * |
| Supplemental motor area | L | -12 | -12 | 63.9 | -4.13344 | * |
| Caudate | - | 0 | 18 | -2.1 | 4.20007 | * |
| Middle temporal gyrus | L | -54 | 0 | -45 | -4.41589 |  |
| Cingulate | - | 0 | -36 | 34.2 | -4.1194 |  |
| Middle temporal gyrus | R | 45 | 3 | -48.3 | -3.69048 |  |
| Precuneus | R | 18 | -42 | 47.4 | 3.80051 |  |
| Anterior cingulate | R | 15 | 45 | -2.1 | 4.25711 | * |
| Middle temporal gyrus | R | 45 | -75 | 21 | 3.26689 |  |
| Cerebellum | L | -12 | -27 | -28.5 | -3.45336 |  |
| Precuneus | L | -6 | -72 | 44.1 | -3.72382 |  |
| Sub-gyral | R | 45 | -9 | -15.3 | 3.58349 |  |
| Hippocampus | R | 36 | -45 | 4.5 | 3.32908 |  |
| Fusiform_R | R | 33 | -42 | -21.9 | -3.73836 |  |
| Postcentral gyrus | R | 21 | -48 | 80.4 | 4.07876 |  |
| Parahippocampus | L | -42 | -27 | -12 | 3.75782 |  |
| Fusiform gyrus | R | 33 | -15 | -35.1 | -3.59373 |  |
| Supplemental motor area | R | 12 | -15 | 67.2 | -3.25712 |  |
| Fusiform gyrus | L | -27 | -15 | -35.1 | -3.7668 |  |

**Figures**

Figure S1. Average betas for the relationship between vmPFC activity and goal values in SC (Replication result: T(14)=6.823, p<.001) and NSC participants (Replication result: T(64)=13.457, p<.001) (A) in the original sample and (B) the replication sample. Error bars represent standard errors.

Figure S2. A) left DLPFC region identified by Hare and colleagues (2009) and B) Meta-analytically identified left DLPFC region of interest used in the replication study. The map includes all left hemisphere voxels of the [www.neurosynth.org](http://www.neurosynth.org) (Yarkoni et al., 2011) map for the term “self-control” intersected with an anatomical DLPFC map (dlPFC definition based on Gozzi et al., 2009).

Figure S3. A) Original study: Average beta values measuring the correlation in IFG/BA9 and IFG/BA46 in the SC and NSC groups. B) Replication study: Average beta values measuring the correlation between BOLD activity in IFG/BA9 cluster (4 mm sphere around [-45, 45, 1]) and left DLPFC individual peaks (where activaty during presentation of unhealthy food was strongest) in the SC and NSC groups; Error bars represent standard errors.

Figure S4. Distribution of self-control success in the replication study sample. The figure shows the distribution of self-control scores on the metric which we used to group participants into self-controllers and non-self-controllers (i.e. percentage of successful self-control trials out of all trials requiring self-control). The graph suggests that self-control, according to this measure, is not a bimodally distributed. It rather seems that a relatively high number of participants perform very poorly on this measure and the remaining participants are sort of uniformly distributed across the whole range.

**References**

Gozzi, M., Raymont, V., Solomon, J., Koenigs, M., Grafman, J., 2009. Dissociable effects of prefrontal and anterior temporal cortical lesions on stereotypical gender attitudes. Neuropsychologia 47, 2125–2132. https://doi.org/10.1016/j.neuropsychologia.2009.04.002

Yarkoni, T., Poldrack, R.A., Nichols, T.E., Van Essen, D.C., Wager, T.D., 2011. Large-scale automated synthesis of human functional neuroimaging data. Nat. Methods 8, 665. https://doi.org/10.1038/NMETH.1635
